# Supplementary material for: Improved prime editors enable pathogenic allele correction and cancer modelling in adult mice
Source: Nat Commun. 2021 Apr 9;12:2121. doi: 10.1038/s41467-021-22295-w (PMC8035190; doi:10.1038/s41467-021-22295-w)

Supplementary Information

Improved prime editors enable pathogenic allele correction and cancer modelling in adult mice

Pengpeng Liu[1¶], Shun-Qing Liang[2¶], Chunwei Zheng[2], Esther Mintzer[1], Yan G. Zhao[1], Karthikeyan Ponnienselvan[1], Aamir Mir[2], Erik J. Sontheimer[2,3,4], Guangping Gao[5], Terence R. Flotte[5,6], Scot A. Wolfe[1,4]*, Wen Xue[2,3,4]*

Supplementary Table 1. Sequences of pegRNAs and sgRNAs used in this study. All sequences are shown in 5' to 3' orientation.

Supplementary Table 2. Sequences of primers used for pegRNA cloning

Supplementary Table 3. Sequences of primers used for genomic DNA amplification and high throughput sequencing.

Supplementary Table 4. Potential genetic engineered liver cancer models using prime editors.

Supplementary Table 5. In vivo on target analysis at SERPINA1 site by UdiTaS.

Supplementary Sequences 1. Sequences of reporter cell line used in this study

Supplementary Sequences 2. Sequence of backbone plasmid used for pegRNA and nicking sgRNA cloning

Supplementary Sequences 3. Sequences of prime editors

Supplementary Note 1. FACS gating examples for GFP-positive or Cherry-positive cells.

Figure S1

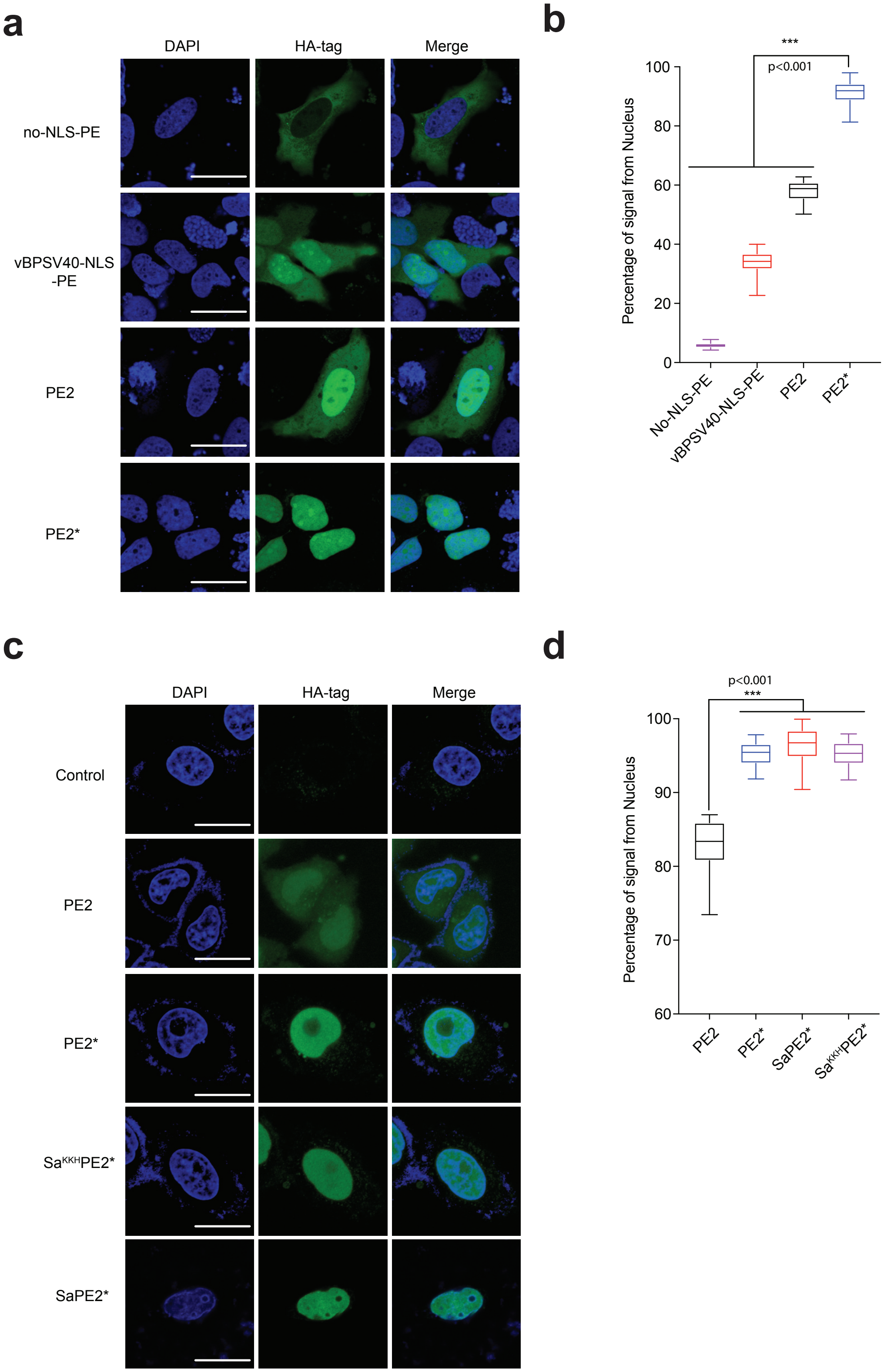
a
DAPI
HA-tag
Merge
no-NLS-PE
vBPSV40-NLS
-PE
PE2
PE2*
b
***
p<0.001
Percentage of signal from Nucleus
100
80
60
40
20
0
No-NLS-PE
vBPSV40-NLS-PE
PE2
PE2*
c
DAPI
HA-tag
Merge
Control
PE2
PE2*
SaKKHPE2*
SaPE2*
d
p<0.001
***
Percentage of signal from Nucleus
100
90
80
70
60
PE2
PE2*
SaPE2*
SaKKHPE2*

Figure S2

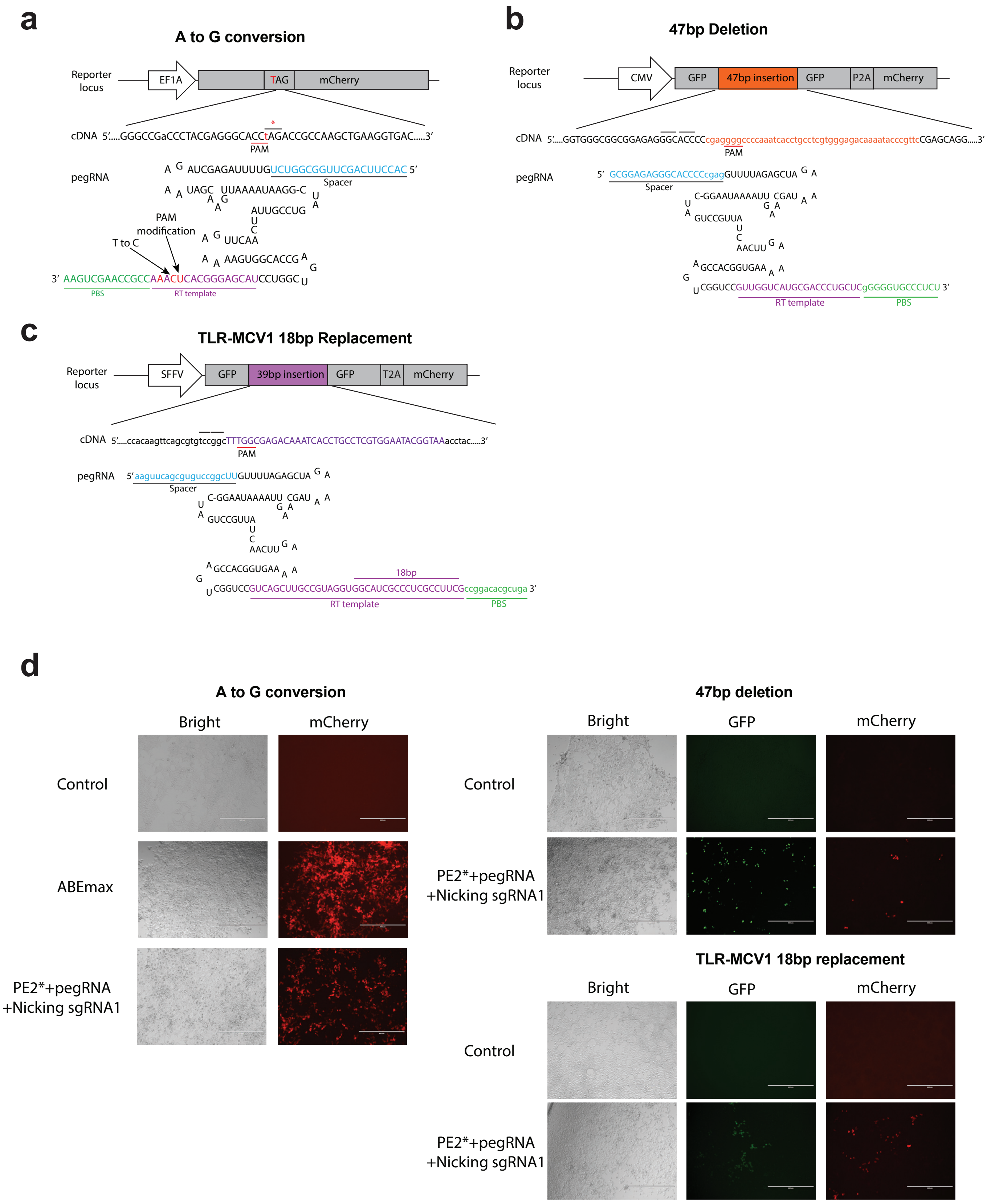
a
A to G conversion
Reporter locus
EF1A
TAG
mCherry
cDNA
PAM
pegRNA
Spacer
PAM modification
T to C
PBS
RT template
b
47bp Deletion
Reporter locus
CMV
GFP
47bp insertion
GFP
P2A
mCherry
cDNA
PAM
pegRNA
Spacer
RT template
PBS
c
TLR-MCV1 18bp Replacement
Reporter locus
SFFV
GFP
39bp insertion
GFP
T2A
mCherry
cDNA
PAM
pegRNA
Spacer
18bp
RT template
PBS
d
A to G conversion
Bright
mCherry
Control
ABEmax
PE2*+pegRNA +Nicking sgRNA1
47bp deletion
Bright
GFP
mCherry
Control
PE2*+pegRNA +Nicking sgRNA1
TLR-MCV1 18bp replacement
Bright
GFP
mCherry
Control
PE2*+pegRNA +Nicking sgRNA1

Figure S3

a

CCR5 Delta32 Deletion

PE2

CAAAAAGAAGGTCTTCATTACACCTGCAGCTCTCATTTTCCATACAGTCAGTATCAATTCTGGAAGAATTTCCAGACATTAAAGATAGTCATCTTGGGGCTGGTCC	WT	94.82%
CAAAAAGAAGGTCTTCATTACACCTGCAGCTCTCATTTTCCtTAtA--------------------------------TTAAAGATAGTCATCTTGGGGCTGGTCC	Precise	3.86%
CAAAAAGAAGGTCTTCATTACACCTGCAGCTCTCATTTTCCA--------------------------------GACATTAAAGATAGTCATCTTGGGGCTGGTCC	-32	0.17%
CAAAAAGAAGGTCTTCATTACACCTGCAGCTCTCATTTTCCtTAtAG--------------------------------TAAAGATAGTCATCTTGGGGCTGGTCC	-32	0.08%
CAAAAAGAAGGTCTTCATTACACCTGCAGCTCTCATTTTCCtTAtA---------------------------------TAAAGATAGTCATCTTGGGGCTGGTCC	-33	0.06%
CAAAAAGAAGGTCTTCATTACACCTGCAGCTCTC------------------ATCAATTCTGGAAGAATTTCCAGACATTAAAGATAGTCATCTTGGGGCTGGTCC	-18	0.04%
CAAAAAGAAGGTCTTCATTACACCTGCAGCTCTCATTTTCCATACAGTCAGTATCAATTCTGGAAGAATTTCCAGAC---AAAGATAGTCATCTTGGGGCTGGTCC	-3	0.03%
CAAAAAGAAGGTCTTCATTACACCTGCAGCTCTCATTTTCCATACAGTCAGTATCAATTCTGGAAGAATTTCCAGACcATTAAAGATAGTCATCTTGGGGCTGGTCC	+1	0.01%

PE2*

CAAAAAGAAGGTCTTCATTACACCTGCAGCTCTCATTTTCCATACAGTCAGTATCAATTCTGGAAGAATTTCCAGACATTAAAGATAGTCATCTTGGGGCTGGTCC	WT	91.25%
CAAAAAGAAGGTCTTCATTACACCTGCAGCTCTCATTTTCCtTAtA--------------------------------TTAAAGATAGTCATCTTGGGGCTGGTCC	Precise	6.08%
CAAAAAGAAGGTCTTCATTACACCTGCAGCTCTCATTTTCCA--------------------------------GACATTAAAGATAGTCATCTTGGGGCTGGTCC	-32	0.25%
CAAAAAGAAGGTCTTCATTACACCTGCAGCTCTCATTTTCCtTAtAG--------------------------------TAAAGATAGTCATCTTGGGGCTGGTCC	-32	0.14%
CAAAAAGAAGGTCTTCATTACACCTGCAGCTCTCATTTT-----------------------------------GACATTAAAGATAGTCATCTTGGGGCTGGTCC	-33	0.08%
CAAAAAGAAGGTCTTCATTACACCTGCAGCTCTCATTTTCCtTAtA---------------------------------TAAAGATAGTCATCTTGGGGCTGGTCC	-33	0.06%
CAAAAAGAAGGTCTTCATTACACCTGCAGCTCTC------------------ATCAATTCTGGAAGAATTTCCAGACATTAAAGATAGTCATCTTGGGGCTGGTCC	-18	0.06%
CAAAAAGAAGGTCTTCATTACACCTGCAGCTCTCATTTTCCATACAGTCAGTATCAATTCTGGAAGAATTTCCAGAC---AAAGATAGTCATCTTGGGGCTGGTCC	-3	0.04%
CAAAAAGAAGGTCTTCATTACACCTGCAGCTCTCATTT--------GTCAGTATCAATTCTGGAAGAATTTCCAGACATTAAAGATAGTCATCTTGGGGCTGGTCC	-8	0,03%
CAAAAAGAAGGTCTTCATTACACCTGCAGCTCTCATTTTCCATACAGTCAGTATCAATTCTGGAAGAATTTCCAGACcATTAAAGATAGTCATCTTGGGGCTGGTCC	+1	0.03%

Sa^{KKH}PE2*

CAAAAAGAAGGTCTTCATTACACCTGCAGCTCTCATTTTCCATACAGTCAGTATCAATTCTGGAAGAATTTCCAGACATTAAAGATAGTCATCTTGGGGCTGGTCC	WT	96.36%
CAAAAAGAAGGTCTTCATTACACCTGCAGCTCTCATTTTCCATACA--------------------------------TTAAAGATAGTCATCTTGGGGCTGGTCC	Precise	2.85%
CAAAAAGAAGGTCTTCATTACACCTGCAGCTCTCATTTTCCA--------------------------------GACATTAAAGATAGTCATCTTGGGGCTGGTCC	-32	0.06%
CAAAAAGAAGGTCTTCATTACACCTGCAGCTCTCATTTT-----------------------------------GACATTAAAGATAGTCATCTTGGGGCTGGTCC	-33	0.04%
CAAAAAGAAGGTCTTCATTACACCTGCAGCTCTCATTTTCCATACAGTCAGTATCAATTCTGGAAGAATTTCCAGAC---AAAGATAGTCATCTTGGGGCTGGTCC	-3	0.02%
CAAAAAGAAGGTCTTCATTACACCTGCAGCTCTCATTTTCCATACAGTCAGTATCAATTCTGGAAGAATTTCCAGAC-----------TCATCTTGGGGCTGGTCC	-11	0.02%
CAAAAAGAAGGTCTTCATTACACCTGCAGCTCTCATTTTCCATACAGTCAGTATCAATTCTGGAAGAA-----------------TAGTCATCTTGGGGCTGGTCC	-17	0.01%

Figure S4

a

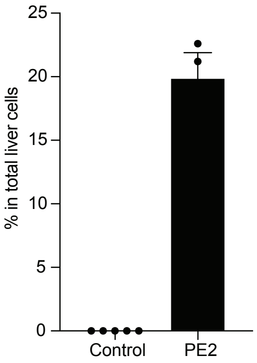

b

K342E correction (via A•T-to-G•C in mice)

PAM modification | Correction | Nicking sgRNA PAM

PE2

CTCTCCCCTCCAGGCCGTGCATAAGGCTGTGCTGACCATCGACAAGAAAGGGACTGAAGCTGCTGGGGCCATGTTTTTAGAGGCCATACCCATGTCTATCCCCCCCGA	WT	93.8%
CTCTCCCCTCCAGGCCGTGCATAAaGCTGTGCTGACCATCGACgAGAAAGGGACTGAAGCTGCTGGGGCCATGTTTTTAGAGGCCATACCCATGTCTATCCCCCCCGA	Precise	2.87%
CTCTCCCCTCCAGGCCGTGC------------TGACCATCGACAAGAAAGGGACTGAAGCTGCTGGGGCCATGTTTTTAGAGGCCATACCCATGTCTATCCCCCCCGA	-12	0.14%
CTCTCCCCTCCAGGC------------TGTGCTGACCATCGACAAGAAAGGGACTGAAGCTGCTGGGGCCATGTTTTTAGAGGCCATACCCATGTCTATCCCCCCCGA	-12	0.11%
CTCTCCCCTCCAGGCCGTGCATAAaGCTGTGCTGACCATCGACgAGA--------------------GCCATGTTTTTAGAGGCCATACCCATGTCTATCCCCCCCGA	-20	0.05%
CTCTCCCCTCCAGG-------------------------------------GACTGAAGCTGCTGGGGCCATGTTTTTAGAGGCCATACCCATGTCTATCCCCCCCGA	-37	0.05%
CTCTCCCCTCCAGGCCGTG----------TGCTGACCATCGACAAGAAAGGGACTGAAGCTGCTGGGGCCATGTTTTTAGAGGCCATACCCATGTCTATCCCCCCCGA	-10	0.03%
CTCTCCCCTCCAGGCCGTGC--CCCCCCGA	-80	0.03%

PE2*

CTCTCCCCTCCAGGCCGTGCATAAGGCTGTGCTGACCATCGACAAGAAAGGGACTGAAGCTGCTGGGGCCATGTTTTTAGAGGCCATACCCATGTCTATCCCCCCCGA	WT	84.7%
CTCTCCCCTCCAGGCCGTGCATAAaGCTGTGCTGACCATCGACgAGAAAGGGACTGAAGCTGCTGGGGCCATGTTTTTAGAGGCCATACCCATGTCTATCCCCCCCGA	Precise	8.68%
CTCTCCCCTCCAGGCCGTGC------------TGACCATCGACAAGAAAGGGACTGAAGCTGCTGGGGCCATGTTTTTAGAGGCCATACCCATGTCTATCCCCCCCGA	-12	0.81%
CTCTCCCCTCCAGGC------------TGTGCTGACCATCGACAAGAAAGGGACTGAAGCTGCTGGGGCCATGTTTTTAGAGGCCATACCCATGTCTATCCCCCCCGA	-12	0.72%
CTCTCCCCTCCAGGCCGTGC--CCCCCCGA	-80	0.67%
CTCTCCCCTCCAGGCCGTGCATAAaGCTGTGCTGACCATCGACgAGA--------------------GCCATGTTTTTAGAGGCCATACCCATGTCTATCCCCCCCGA	-20	0.43%
CTCTCCCCTCCAGGCC---ATACCCATGTCTATCCCCCCCGA	-69	0.26%
CTCTCCCCTCCAGGCCGTGCcATAAaGCTGTGCTGACCATCGACAAGAAAGGGACTGAAGCTGCTGGGGCCATGTTTTTAGAGGCCATACCCATGTCTATCCCCCCCGA	+1	0.11%
CTCTCCCCTCCAGGCCGTGCATAAGGCTGTGCTGAGCATCGACAAGAAAGGGACTGAAGCTGCTGGGGCCATGTTTTTAGAGGCCATACCCATGTCTATCCCCCCCGA	sub	0.07%
CTCTCCCCTCCAGGCCGTGCATAAGGCTGTGCTGACCATCGACgAGAAAGGGACTGAAGCTGCTGGGGCCATGTTTTTAGAGGCCATACCCATGTCTATCCCCCCCGA	sub	0.06%
CTCTCCCCTCCAGGCCGTGCATAAaGCTGTGCTGACCATCGACAAGAAAGGGACTGAAGCTGCTGGGGCCATGTTTTTAGAGGCCATACCCATGTCTATCCCCCCCGA	sub	0.04%

Figure S5

a

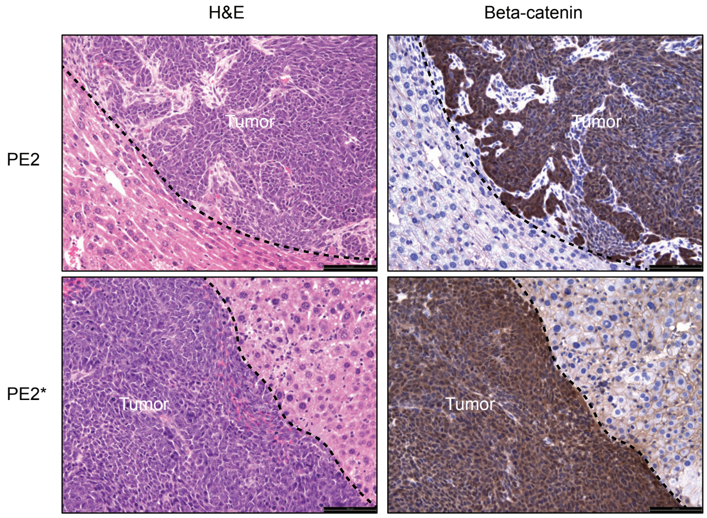

Figure S6

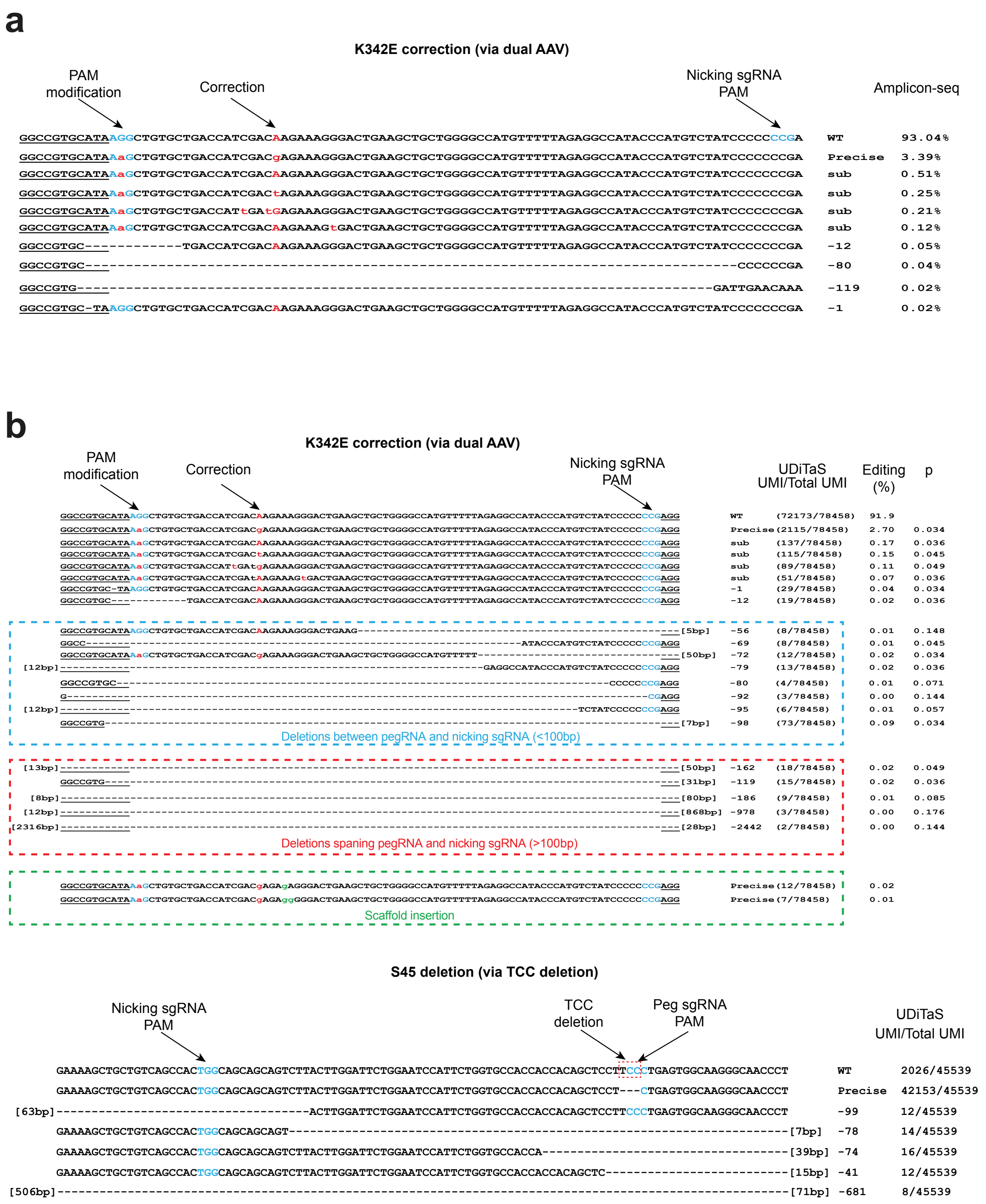
a
K342E correction (via dual AAV)
PAM modification
Correction
Nicking sgRNA PAM
Amplicon-seq
GGCCGTGCATAAGGCTGTGCTGACCATCGACAAGAAAGGGACTGAAGCTGCTGGGGCCATGTTTTTAGAGGCCATACCCATGTCTATCCCCCCCGA WT 93.04%
GGCCGTGCATAAaGCTGTGCTGACCATCGACgAGAAAGGGACTGAAGCTGCTGGGGCCATGTTTTTAGAGGCCATACCCATGTCTATCCCCCCCGA Precise 3.39%
GGCCGTGCATAAaGCTGTGCTGACCATCGACAAGAAAGGGACTGAAGCTGCTGGGGCCATGTTTTTAGAGGCCATACCCATGTCTATCCCCCCCGA sub 0.51%
GGCCGTGCATAAaGCTGTGCTGACCATCGACtAGAAAGGGACTGAAGCTGCTGGGGCCATGTTTTTAGAGGCCATACCCATGTCTATCCCCCCCGA sub 0.25%
GGCCGTGCATAAaGCTGTGCTGACCATtGAtGAGAAAGGGACTGAAGCTGCTGGGGCCATGTTTTTAGAGGCCATACCCATGTCTATCCCCCCCGA sub 0.21%
GGCCGTGCATAAaGCTGTGCTGACCATCGACAAGAAAGtGACTGAAGCTGCTGGGGCCATGTTTTTAGAGGCCATACCCATGTCTATCCCCCCCGA sub 0.12%
GGCCGTGC------------TGACCATCGACAAGAAAGGGACTGAAGCTGCTGGGGCCATGTTTTTAGAGGCCATACCCATGTCTATCCCCCCCGA -12 0.05%
GGCCGTGC--CCCCCCGA -80 0.04%
GGCCGTG--GATTGAACAAA -119 0.02%
GGCCGTGC-TAAGGCTGTGCTGACCATCGACAAGAAAGGGACTGAAGCTGCTGGGGCCATGTTTTTAGAGGCCATACCCATGTCTATCCCCCCCGA -1 0.02%
b
K342E correction (via dual AAV)
PAM modification
Correction
Nicking sgRNA PAM
UDiTaS UMI/Total UMI
Editing (%)
p
GGCCGTGCATAAGGCTGTGCTGACCATCGACAAGAAAGGGACTGAAGCTGCTGGGGCCATGTTTTTAGAGGCCATACCCATGTCTATCCCCCCCGAGG WT (72173/78458) 91.9
GGCCGTGCATAAaGCTGTGCTGACCATCGACgAGAAAGGGACTGAAGCTGCTGGGGCCATGTTTTTAGAGGCCATACCCATGTCTATCCCCCCCGAGG Precise (2115/78458) 2.70 0.034
GGCCGTGCATAAaGCTGTGCTGACCATCGACAAGAAAGGGACTGAAGCTGCTGGGGCCATGTTTTTAGAGGCCATACCCATGTCTATCCCCCCCGAGG sub (137/78458) 0.17 0.036
GGCCGTGCATAAaGCTGTGCTGACCATCGACtAGAAAGGGACTGAAGCTGCTGGGGCCATGTTTTTAGAGGCCATACCCATGTCTATCCCCCCCGAGG sub (115/78458) 0.15 0.045
GGCCGTGCATAAaGCTGTGCTGACCATtGAtgAGAAAGGGACTGAAGCTGCTGGGGCCATGTTTTTAGAGGCCATACCCATGTCTATCCCCCCCGAGG sub (89/78458) 0.11 0.049
GGCCGTGCATAAaGCTGTGCTGACCATCGAtAAGAAAGtGACTGAAGCTGCTGGGGCCATGTTTTTAGAGGCCATACCCATGTCTATCCCCCCCGAGG sub (51/78458) 0.07 0.036
GGCCGTGC-TAAGGCTGTGCTGACCATCGACAAGAAAGGGACTGAAGCTGCTGGGGCCATGTTTTTAGAGGCCATACCCATGTCTATCCCCCCCGAGG -1 (29/78458) 0.04 0.034
GGCCGTGC------------TGACCATCGACAAGAAAGGGACTGAAGCTGCTGGGGCCATGTTTTTAGAGGCCATACCCATGTCTATCCCCCCCGAGG -12 (19/78458) 0.02 0.036
GGCCGTGCATAAGGCTGTGCTGACCATCGACAAGAAAGGGACTGAAG---[5bp] -56 (8/78458) 0.01 0.148
GGCC---ATACCCATGTCTATCCCCCCCGAGG -69 (8/78458) 0.01 0.045
GGCCGTGCATAAaGCTGTGCTGACCATCGACgAGAAAGGGACTGAAGCTGCTGGGGCCATGTTTTT---------------------------------[50bp] -72 (12/78458) 0.02 0.034
[12bp]--GAGGCCATACCCATGTCTATCCCCCCCGAGG -79 (13/78458) 0.02 0.036
GGCCGTGC--CCCCCCGAGG -80 (4/78458) 0.01 0.071
G--CGAGG -92 (3/78458) 0.00 0.144
[12bp]--TCTATCCCCCCCGAGG -95 (6/78458) 0.01 0.057
GGCCGTG--[7bp] -98 (73/78458) 0.09 0.034
Deletions between pegRNA and nicking sgRNA (<100bp)
[13bp]--[50bp] -162 (18/78458) 0.02 0.049
GGCCGTG--[31bp] -119 (15/78458) 0.02 0.036
[8bp]---[80bp] -186 (9/78458) 0.01 0.085
[12bp]--[868bp] -978 (3/78458) 0.00 0.176
[2316bp]--[28bp] -2442 (2/78458) 0.00 0.144
Deletions spaning pegRNA and nicking sgRNA (>100bp)
GGCCGTGCATAAaGCTGTGCTGACCATCGACgAGAgAGGGACTGAAGCTGCTGGGGCCATGTTTTTAGAGGCCATACCCATGTCTATCCCCCCCGAGG Precise (12/78458) 0.02
GGCCGTGCATAAaGCTGTGCTGACCATCGACgAGAggGGGACTGAAGCTGCTGGGGCCATGTTTTTAGAGGCCATACCCATGTCTATCCCCCCCGAGG Precise (7/78458) 0.01
Scaffold insertion
S45 deletion (via TCC deletion)
Nicking sgRNA PAM
TCC deletion
Peg sgRNA PAM
UDiTaS UMI/Total UMI
GAAAAGCTGCTGTCAGCCACTGGCAGCAGCAGTCTTACTTGGATTCTGGAATCCATTCTGGTGCCACCACCACAGCTCCTTCCCTGAGTGGCAAGGGCAACCCT WT 2026/45539
GAAAAGCTGCTGTCAGCCACTGGCAGCAGCAGTCTTACTTGGATTCTGGAATCCATTCTGGTGCCACCACCACAGCTCCT---CTGAGTGGCAAGGGCAACCCT Precise 42153/45539
[63bp]------------------------------------ACTTGGATTCTGGAATCCATTCTGGTGCCACCACCACAGCTCCTTCCCTGAGTGGCAAGGGCAACCCT -99 12/45539
GAAAAGCTGCTGTCAGCCACTGGCAGCAGCAGT--[7bp] -78 14/45539
GAAAAGCTGCTGTCAGCCACTGGCAGCAGCAGTCTTACTTGGATTCTGGAATCCATTCTGGTGCCACCA----------------------------------[39bp] -74 16/45539
GAAAAGCTGCTGTCAGCCACTGGCAGCAGCAGTCTTACTTGGATTCTGGAATCCATTCTGGTGCCACCACCACAGCTC-------------------------[15bp] -41 12/45539
[506bp]--[71bp] -681 8/45539

Supplementary Fig. 1 Optimized prime editor increases the degree of nuclear import. (a) Representative immunofluorescence images of prime editors transfected into U2OS cells immunostained with a HA tag antibody to visualize subcellular localization. PE2 contains two Bipartite SV40 NLSs, whereas the PE2* variant include an extra N-terminal c-Myc NLS and a C-terminal variant bipartite SV40 NLS (vBPSV40) and SV40. DNA was stained with DAPI. (b) Average percentages of HA-tag signal from nucleus for different prime editors. (c-d) Representative immunofluorescence images and quantification in HeLa cells. For (b) and (d), >90 cells were analyzed for each group from 3 independent experiments. ****P*<0.001 by one-way ANOVA with Tukey's multiple comparisons test. In the boxes, the top, middle and bottom lines represent the 25, 50 and 75 percentiles, respectively. Whiskers indicated the min and max percentiles and outliers are not shown. Scale bars, 10µm.

Supplementary Fig. 2 Prime editing in reporter cells by PE2 and PE2*. (a) Sequence of reporter locus and pegRNA used for repair of mCherry reporter in HEK293T cells. Bar above the cDNA indicates the stop codon with the target "t" for conversion indicated in red. Two additional silent mutations are included to discourage recutting of the repaired DNA sequence. (b) Sequence of reporter and pegRNA used for generation of a 47bp deletion to restore function of the GFP reporter in HEK293T cells. The bars above the cDNA indicates three nucleotide blocks that correspond to codons in the GFP reporter. (c) Sequence of reporter and pegRNA used for replacement of a 18bp element to restore function of the GFP reporter. The bars above the cDNA indicates three nucleotide blocks that correspond to codons in the GFP reporter. (d) Representative images of HEK293T reporter cells (n=3) transfected with control, ABEmax or PE2*. Scale bar, 400µm.

Supplementary Fig.3 Sequencing analysis of the *CCR5* prime editing by PE2, PE2*, or Sa^{KKH}PE2*.
The percentage of most common sequences in PE2, PE2* and Sa^{KKH}PE2* transfected cells is shown on the right (representative of n=3, determined by Illumina sequencing). The PE target site is underlined. The PAM sequences are indicated in light blue for SpCas9 and green for SaCas9^{KKH}. The box denotes the 32bp sequence deleted in CCR5delta32. Two "t" mutations in red were included in the PE2 and PE2* RT template (Fig. 2d) to demonstrate that sequence collapse was not a function of nuclease-induced microhomology mediated deletion and to reduce re-cutting of deletion allele. Deleted bases are indicated by dashes and inserted bases are in blue.

Supplementary Fig.4 Sequencing analysis of *SERPINA1* editing in the liver of PiZ mouse.
(a) Evaluating prime editor expression in mouse liver. FVB mice were injected with 30µg of control vector or PE2 plasmid with an incorporated 3xHA-tag. Livers were harvested at day 2 and IHC staining were performed with an HA-tag antibody. Quantification of HA^{+} cells. Numbers are mean ± sem (n=5 images from 4 mice for each group).
(b) The percentage of most common sequences in the liver of PE2 and PE2*-treated mice is shown on the right (representative liver of n=3, determined by Illumina sequencing). The PE target site is underlined. The PAM sequences are in light blue. Nucleotide substitutions are labeled in red. Deleted bases are indicated by dashes. Inserted bases are shown in blue/lower case.

Supplementary Fig.5 Liver tumors are positive for nuclear beta-Catenin. (a) Representative H&E and beta-catenin IHC staining (n=3) in PE2 or PE2*-induced S45F tumors in Fig. 4. Scale bars: 100 µm (20X lens).

Supplementary Fig. 6 Sequencing analysis of the *Ctnnb1* and *SERPINA1* prime editing by PE2 or PE2*. (a) The percentage of most common sequences in the liver of dual AAV-treated PiZ mice determined by amplicon sequencing is shown on the right (representative liver of n=3). A portion of the PE target site is underlined. The PAM sequences are in light blue. Nucleotide substitutions are labeled in red. Deleted bases are indicated by dashes. (b) Length distribution of precise editing and other indels at *SERPINA1 (K342E correction)* and *Ctnnb1* target sites by UDiTaS. Also included are sequence modifications that may be associated with pegRNA scaffold insertions[7]. Data for a representative liver per group is shown (n=3 mice).

Supplementary Table 1. Sequences of pegRNAs and sgRNAs used in this study. All sequences are shown in 5' to 3' orientation.

sgRNA scaffold

GTTTTAGAGCTAGAAATAGCAAGTTAAAATAAGGCTAGTCCGTTATCAACTTGAAAAAGTGGGACCGAGTCGGTCC

**pegRNA**	**spacer sequence (5'-3')**	**3' extension**	**PBS (nt)**	**RT (nt)**	**Figure**	**PE**
mCherry A to G	CACCTTCAGCTTGGCGGTCT	TACGAGGGCACTCAAACCGCCAAGCTGAAG	14	16	Figure 1B	PE2 or PE2*
mCherry A to G	GGTCACCTTCAGCTTGGCGGT	TACGAGGGCACCCAGACTGCCAAGCTGAAGGTGA	16	17	Figure 1B	$Sa^{KKH}PE2^*$
GFP-insertion	AAGTTCAGCGTGTCCGGCTT	GTCAGCTTGCCGTAGGTGGCATCGCCCTCGCCTTCG	13	36	Figure 1C	PE2 or PE2*
GFP-insertion	TGAACTTCAGGGTCAGCTTGCC	ACAAGTTCAGCGTGTCCGGCGAGGGCGAGGGCGATGCCACGTACGGCAAGCTGACCCTGAAGTTC	18	47	Figure 1C	$Sa^{KKH}PE2^*$
GFP-deletion	GCGGAGAGGGCACCCCCGA	GTTGGTCATGCGACCCTGCTCGGGGGTGCCCTCTCC	14	22	Figure 1D	PE2 or PE2*
GFP-deletion	GGTCATGCGACCCTGCTCGGA	CGGAGAGGGCACCCCCGAGCAGGGTCGCATG	15	16	Figure 1D	$Sa^{KKH}PE2^*$
EMX1 +5 G to T	GAGTCCGAGCAGAAGAAGAA	ATGGGAGCACTTCTTCTTCTGCTC	14	10	Figure 2A	PE2 or PE2*
EMX1 +3 +8 G to T	CAGAAGCTGGAGGAGGAAGGGC	TGCTCGGAATCAGACCCTTCCTCCTCCAGCT	15	16	Figure 2A	SaCas9 PE2* or $Sa^{KKH}PE2^*$
EMX1 +4 3bp deletion	GAGTCCGAGCAGAAGAAGAA	ATGTGATGGGAGTTCTTCTTCTGCTC	14	12	Figure 2B	PE2 or PE2*
EMX1 +1 3bp deletion	CAGAAGCTGGAGGAGGAAGGGC	TTCTGCTCGGACTCAGCTTCCTCCTCCAGCT	15	16	Figure 2B	SaCas9 PE2* or $Sa^{KKH}PE2^*$
EMX1 +4 6bp Insertion	GAGTCCGAGCAGAAGAAGAA	GAGCAGAAGAAGAAAAGCTTGGGCTCCCATCACAT	14	21	Figure 2C	PE2 or PE2*
EMX1 +1 6bp Insertion	CAGAAGCTGGAGGAGGAAGGGC	TGCTCGGACTCAGGCCAAGCTTCTTCCTCCTCCAGCT	15	22	Figure 2C	SaCas9 PE2* or $Sa^{KKH}PE2^*$
CCR5-delta32-deletion	AGATGACTATCTTTAATGTC	ATTACACCTGCAGCTCTCATTTTCCTTATATTAAAGATAGTCATC	16	29	Figure 2D	PE2 or PE2*
CCR5-delta32-deletion	AAGATGACTATCTTTAATGTCT	AGCTCTCATTTTCCATACATTAAAGATAGTCATC	17	17	Figure 2D	$Sa^{KKH}PE2^*$
Serpina1 G to A	TCCCCTCCAGGCCGTGCATA	TCTTGTCGATGGTCAGCACAGCTTTATGCACGGCCTGGAG	13	27	Figure 3A	PE2 or PE2*
Serpina1 G to A	CAGCTTCAGTCCCTTTCTCGT	ATCGACAAGAAAGGGACTGAAGCT	15	9	Figure 3A	$Sa^{KKH}PE2^*$
Serpina1 A to G	TCCCCTCCAGGCCGTGCATA	TCTCGTCGATGGTCAGCACAGCTTTATGCACGGCCTGGAG	13	27	Figure 3B	PE2 or PE2*
Ctnnb1 C to T	AGGGTTGCCCTTGCCACTCA	GCTCCTTTCCTGAGTGGCAAGGGCAA	13	13	Figure 4A	PE2 or PE2*
Ctnnb1 C to T	AGGGTTGCCCTTGCCACTCA	ACAGCTCCTTTGAGTGGCAAGGGCAA	13	13	Figure 4D	PE2*

**Nicking sgRNA**	**spacer sequence (5'-3')**	**Figure**	**PE**
mCherry A to G	GCGCTTCAAGGTGCACATGGA	Figure 1B	PE2 or PE2*
mCherry A to G	GCTGTCCCCTCAGTTCATGTA	Figure 1B	PE2 or PE2*
mCherry A to G	GATGGAGGGCTCCGTGAACGGCC	Figure 1B	$Sa^{KKH}PE2^*$
mCherry A to G	GTTCGCCTGGGACATCCTGTCCC	Figure 1B	$Sa^{KKH}PE2^*$
GFP-insertion-sp-NK1	GTAGGTCAGGGTGGTCACGA	Figure 1C	PE2 or PE2*
GFP-insertion-sp-NK2	GCTCCTCGCCCTTGCTCACCA	Figure 1C	PE2 or PE2*
GFP-insertion-saKKH-NK1	GCAAGGGCGAGGAGCTGTTCAC	Figure 1C	$Sa^{KKH}PE2^*$

GFP-insertion-saKKH-NK2	GTGACCACCCTGACCTACGGCG	Figure 1C	$Sa^{KKH}PE2*$
GFP-deletion-sp-NK1	GAGAAGCCGTAGCCCATCACG	Figure 1D	PE2 or PE2*
GFP-deletion-sp-NK2	GATCTTCATGGCGGGCATGG	Figure 1D	PE2 or PE2*
GFP-deletion-saKKH-NK1	GATCACCGGCACCCTGAACGGCG	Figure 1D	$Sa^{KKH}PE2*$
GFP-deletion-saKKH-NK2	GCAGCCCCTACCTGCTGAGCCA	Figure 1D	$Sa^{KKH}PE2*$
EMX1 +5 G to T-sp-NK1	GTTGCCCACCCTAGTCATTGG	Figure 2A	PE2 or PE2*
EMX1 +5 G to T-sp-NK2	GGCCGTTTGTACTTTGTCCTC	Figure 2A	PE2 or PE2*
EMX1 +3 +8 G to T-sa-NK1	GCCTGGGCCAGGGAGGGAGGGGC	Figure 2A	SaCas9 PE2* or $Sa^{KKH}PE2*$
EMX1 +3 +8 G to T-sa-NK2	GTGGTTGCCCACCCTAGTCATT	Figure 2A	SaCas9 PE2* or $Sa^{KKH}PE2*$
EMX1 +4 3bp deletion-sp-NK1	GTTGCCCACCCTAGTCATTGG	Figure 2B	PE2 or PE2*
EMX1 +4 3bp deletion-sp-NK2	GGCCGTTTGTACTTTGTCCTC	Figure 2B	PE2 or PE2*
EMX1 +1 3bp deletion-sa-NK1	GCCTGGGCCAGGGAGGGAGGGGC	Figure 2B	SaCas9 PE2* or $Sa^{KKH}PE2*$
EMX1 +1 3bp deletion-sa-NK2	GTGGTTGCCCACCCTAGTCATT	Figure 2B	SaCas9 PE2* or $Sa^{KKH}PE2*$
EMX1 +4 6bp Insertion-sp-NK1	GTTGCCCACCCTAGTCATTGG	Figure 2C	PE2 or PE2*
EMX1 +4 6bp Insertion-sp-NK2	GGCCGTTTGTACTTTGTCCTC	Figure 2C	PE2 or PE2*
EMX1 +1 6bp Insertion-sa-NK1	GCCTGGGCCAGGGAGGGAGGGGC	Figure 2C	SaCas9 PE2* or $Sa^{KKH}PE2*$
EMX1 +1 6bp Insertion-sa-NK2	GTGGTTGCCCACCCTAGTCATT	Figure 2C	SaCas9 PE2* or $Sa^{KKH}PE2*$
CCR5-deletion-sp-NK1	GCTGTGTTTGCGTCTCTCCC	Figure 2E	PE2 or PE2*
CCR5-deletion-sp-NK2	GACAAGTGTGATCACTTGGG	Figure 2E	PE2 or PE2*
CCR5-deletion-sp-NK3	GCAGGACGGTCACCTTTGGGG	Figure 2E	PE2 or PE2*
CCR5-deletion-saKKH-NK1	GCATCTTTACCAGATCTCAAAAA	Figure 2E	$Sa^{KKH}PE2*$
CCR5-deletion-saKKH-NK2	GTGGCTGTGTTTGCGTCTCTCCC	Figure 2E	$Sa^{KKH}PE2*$
Serpina1 G to A-sp-NK1	GGGGGGGATAGACATGGGTA	Figure 3A	PE2 or PE2*
Serpina1 G to A-sp-NK2	GACCTCGGGGGGGATAGACA	Figure 3A	PE2 or PE2*
Serpina1 G to A-sp-NK3	GGGTTTGTTGAACTTGACCT	Figure 3A	PE2 or PE2*
Serpina1 G to A-sp-NK4	GTTCAATCATTAAGAAGACAA	Figure 3A	PE2 or PE2*
Serpina1 G to A	GCACGTGAGCCTTGCTCGAGGCC	Figure 3A	$Sa^{KKH}PE2*$
Serpina1 G to A	GCCCATGTCTATCCCCCCCGAGG	Figure 3A	$Sa^{KKH}PE2*$
Serpina1 A to G (mouse_injection)	GGGTTTGTTGAACTTGACCT	Figure 3B	PE2 or PE2*
Ctnnb1 C to T	GAAAAGCTGCTGTCAGCCAC	Figure 4A	PE2 or PE2*

Supplementary Table 2. Sequences of primers used for pegRNA cloning

pegRNA	Sequence (5'-3')	Figure	PE
mCherry A to G_F	TATCTTGTGGAAAGGACGAAACACCGCACCTTCAGCTTGGCGGTCTGTTTTA GAGCTAGAAATAG	Figure 1B	PE2 or PE2*
mCherry A to G_R	CGGTATCGATAAGCTTGATATCGAATTCAAAAAACTTCAGCTTGGCGGTTTG AGTGCCCTCGTAGGACCGACTCGGTCCCACTTTTTC	Figure 1B	PE2 or PE2*
mCherry A to G_F	TATCTTGTGGAAGGACGAAACACCGGTCACCTTCAGCTTGGCGGTGTTTTAG TACTCTGGAAACAG	Figure 1B	$Sa^{KKH}PE2*$
mCherry A to G_R	CGACGGTATCGATAAGCTTGATATCGAATTCAAAAAAATCACCTTCAGCTTG GCAGTCTGGGTGCCCTCGTATCTCGCCAACAAGTTGACGAGAT	Figure 1B	$Sa^{KKH}PE2*$
GFP-insertion_F	TGGAAAGGACGAAACACCGAAGTTCAGCGTGTCCGGCTTGTTTTAGAGCTAG AAATAG	Figure 1C	PE2 or PE2*
GFP-insertion_R	GTATCGATAAGCTTGATATCAAAAAATCAGCGTGTCCGGCGAAGGCGAGGGC GATGCCACCTACGGCAAGCTGACGGACCGACTCGGTCCCACTTTTTC	Figure 1C	PE2 or PE2*
GFP-insertion_F	GTGGAAGGACGAAACACCGTGAACTTCAGGGTCAGCTTGCCGTTTTAGTACT CTGGAAACAGA	Figure 1C	$Sa^{KKH}PE2*$

GFP-insertion_R	ATCTCGTCAACTTGTTGGCGAGAACAAGTTCAGCGTGTCCGGCGAGGGCGAGGGCGATGCCACGTACGGCAAGCTGACCCTGAAGTTCTTTTTTTGAATTCGATATCAAGCTTATCGATACCGTCG	Figure 1C	Sa^{KKH}PE2*
GFP-deletion_F	TGGAAAGGACGAAACACCGGCGGAGAGGGCACCCCCGAGTTTTAGAGCTAGAAATAG	Figure 1D	PE2 or PE2*
GFP-deletion_R	GTATCGATAAGCTTGATATCAAAAAAGGAGAGGGCACCCCCGAGCAGGGTCGCATGACCAACGGACCGACTCGGTCCCACTTTTTCAAG	Figure 1D	PE2 or PE2*
GFP-deletion_F	GTGGAAGGACGAAACACCGGGTCATGCGACCCTGCTCGGAGTTTTAGTACTCTGGAAACAG	Figure 1D	Sa^{KKH}PE2*
GFP-deletion_R	CGACGGTATCGATAAGCTTGATATCGAATTCAAAAAAACATGCGACCCTGCTCGGGGGTGCCCTCTCCGTCTCGCCAACAAGTTGACGAGAT	Figure 1D	Sa^{KKH}PE2*
EMX1 +5 G to T-sp_F	TGGAAAGGACGAAACACCGGAGTCCGAGCAGAAGAAGAAGTTTTAGAGCTAGAAATAG	Figure 2A	PE2 or PE2*
EMX1 +5 G to T-sp_R	GTATCGATAAGCTTGATATCAAAAAAGAGCAGAAGAAGAAGTGCTCCCATGGACCGACTCGGTCCCACTTTTTC	Figure 2A	PE2 or PE2*
EMX1 +3 +8 G to T-sa_F	GTGGAAGGACGAAACACCGCAGAAGCTGGAGGAGGAAGGGCGTTTTAGTACTCTGGAAACAG	Figure 2A	SaCas9 PE2* or Sa^{KKH}PE2*
EMX1 +3 +8 G to T-sa_R	CGACGGTATCGATAAGCTTGATATCGAATTCAAAAAAAAGCTGGAGGAGGAAGGGTCTGATTCCGAGCATCTCGCCAACAAGTTGACGAGAT	Figure 2A	SaCas9 PE2* or Sa^{KKH}PE2*
EMX1 +4 3bp deletion-sp_F	TGGAAAGGACGAAACACCGGAGTCCGAGCAGAAGAAGAAGTTTTAGAGCTAGAAATAG	Figure 2B	PE2 or PE2*
EMX1 +4 3bp deletion-sp_R	GTATCGATAAGCTTGATATCAAAAAAGAGCAGAAGAAGAACTCCCATCACATGGACCGACTCGGTCCCACTTTTTC	Figure 2B	PE2 or PE2*
EMX1 +1 3bp deletion-sa_F	GTGGAAGGACGAAACACCGCAGAAGCTGGAGGAGGAAGGGCGTTTTAGTACTCTGGAAACAG	Figure 2B	SaCas9 PE2* or Sa^{KKH}PE2*
EMX1 +1 3bp deletion-sa_R	CGACGGTATCGATAAGCTTGATATCGAATTCAAAAAAAAGCTGGAGGAGGAAGCTGAGTCCGAGCAGAATCTCGCCAACAAGTTGACGAGAT	Figure 2B	SaCas9 PE2* or Sa^{KKH}PE2*
EMX1 +4 6bp Insertion-sp_F	TGGAAAGGACGAAACACCGGAGTCCGAGCAGAAGAAGAAGTTTTAGAGCTAGAAATAG	Figure 2C	PE2 or PE2*
EMX1 +4 6bp Insertion-sp_R	GTATCGATAAGCTTGATATCAAAAAAGAGCAGAAGAAGAAAAGCTTGGGCTCCCATCACATGGACCGACTCGGTCCCACTTTTTC	Figure 2C	PE2 or PE2*
EMX1 +1 6bp Insertion-sa_F	GTGGAAGGACGAAACACCGCAGAAGCTGGAGGAGGAAGGGCGTTTTAGTACTCTGGAAACAG	Figure 2C	SaCas9 PE2* or Sa^{KKH}PE2*
EMX1 +1 6bp Insertion-sa_R	CGACGGTATCGATAAGCTTGATATCGAATTCAAAAAAAAGCTGGAGGAGGAAGAAGCTTGGCCTGAGTCCGAGCATCTCGCCAACAAGTTGACGAGAT	Figure 2C	SaCas9 PE2* or Sa^{KKH}PE2*
CCR5-deletion-sp_F	TGGAAAGGACGAAACACCGAGATGACTATCTTTAATGTCGTTTTAGAGCTAGAAATAG	Figure 2E	PE2 or PE2*
CCR5-deletion-sp_R	GTATCGATAAGCTTGATATCAAAAAAGATGACTATCTTTAATATAAGGAAAATGAGAGCTGCAGGTGGACCGACTCGGTCCCACTTTTTC	Figure 2E	PE2 or PE2*
CCR5-deletion-saKKH_F	GTGGAAGGACGAAACACCGAAGATGACTATCTTTAATGTCTGTTTTAGTACTCTGGAAACAG	Figure 2E	Sa^{KKH}PE2*
CCR5-deletion-saKKH_R	GTATCGATAAGCTTGATATCAAAAAAGATGACTATCTTTAATATAAGGAAAATGAGAGCTGCAGGTGTAATGGACCGACTCGGTCCCACTTTTTC	Figure 2E	Sa^{KKH}PE2*
Serpina1 G to A gblocks	TATATATCTTGTGGAAAGGACGAAACACCGtTATATATCTTGTGGAAAGGACGAAACACCGtcccctccaggccgtgcatagttttagagctagaaatagcaagttaaaataaggctagtccgttatcaacttg	Figure 3A	PE2 or PE2*
Serpina1 G to A gblocks	TATATATCTTGTGGAAAGGACGAAACACCGcaTATATATCTTGTGGAAAGGACGAAACACCGtcccctccaggccgtgcatagttttagagctagaaatagcaagttaaaataaggctagtccgttatcaacttg	Figure 3A	Sa^{KKH}PE2*
Serpina1 A to G gblocks	TATATATCTTGTGGAAAGGACGAAACACCGTATATATCTTGTGGAAAGGACGAAACACCGtcccctccaggccgtgcatagttttagagctagaaatagcaagttaaaataaggctagtccgttatcaacttg	Figure 3B	PE2 or PE2*
Ctnnb1 C to T gblocks	ATCTTGTGGAAAGGACGAAACACCGAGGGTTGCCCTTGCCACTCAgttttagagctagaaatagcaagttaaaataaggctagtccgttatcaacttgaaaaagtgggaccgagtcggtccGCTCCTTTCCTGAGTGGCAAGGGCAATTTTTTTGAATTCGATATCAAGCTTATCGATACCGT	Figure 4A	PE2 or PE2*

Supplementary Table 3. Sequences of primers used for genomic DNA amplification and high throughput sequencing.

Figure	Description	PE		Squence
Figure 2A~2C	EMX1_locus	PE2/PE2* or SaCas9	5p-DS1_EMX1	ctacacgacgctcttccgatctCCTCCTGAGTTTCTCATCTGTGCC
Figure 2A~2C			3p-DS1_EMX1	agacgtgtgctcttccgatctTCTGCCCTCGTGGGTTTGTG

Figure 2A~2C		PE2*/SaKKH PE2*	5p-DS2_EMX1	ctacacgacgctcttccgatctGGACAAAGTA CAAACGGCAGAAGC
Figure 2A~2C			3p-DS2_EMX1	agacgtgtgctcttccgatctCAGCCAGCCCA TTGCTTGTC
Figure 2D~2E	CCR5-deletion	PE2/PE2* or SaKKHPE2*	DS-5P-CR5_UMI-deletion	CTACACGACGCTCTTCCGATCTNNWNNVHBGT CTCTCCCAGGAATCATCTTTACCAG
Figure 2D~2E			3p_DS_constant	AGACGTGTGCTCTTCCGAT
Figure 2D~2E			5p-DS_CCR5	ctacacgacgctcttccgatctGTCTCTCCCA GGAATCATCTTTACCAG
Figure 2D~2E			3p-DS_CCR5	agacgtgtgctcttccgatctCGACACCGAAG CAGAGTTTTTAGGAT
Figure 3A~3D	Serpina1 G to A or Serpina1 A to G	PE2/PE2* or SaKKHPE2*	5p-AAT-DS	ctacacgacgctcttccgatctATAAGGCTGT GCTGACCATCG
Figure 3A~3D				
Figure 3A~3D				
Figure 3A~3D			3p-AAT-DS	agacgtgtgctcttccgatctGGGGAGACTTG GTATTTTGTTCA
Figure 3A~3D				
Figure 3A~3D				
Figure 4	Ctnnb1 TCC deletion	PE2/PE2*	ctnnb1-Uditas-FWD	GTGACTGGAGTTCAGACGTGTGCTCTTCCGAT CTGCTTCTTCAGGTAGCATTTTCAGTTCAC
Figure 4			ctnnb1-Uditas-REV	GTGACTGGAGTTCAGACGTGTGCTCTTCCGAT CTGCTTCCAAACACAAATGCTTTACCAG
Figure 5	Serpina1 A to G	AAV	SerpinA1-Uditas-FWD	GTGACTGGAGTTCAGACGTGTGCTCTTCCGAT CTagccttacaacgtgtctctgcttc
Figure 5			SerpinA1-Uditas-REV	GTGACTGGAGTTCAGACGTGTGCTCTTCCGAT CTgcagttatttttgggtgggattca

Supplementary Table 4. Potential genetic engineered liver cancer models using prime editors.

Strain	Target gene	Accompanying gene/mutation
FVB/B6	p53 R270H	c-Myc
FVB/B6	HrasG12V	c-Myc
FVB/B6	NrasG12V	c-Myc
FVB/B6	KrasG12V	p53 R270H
FVB/B6	Ctnnb1 S45F	YapS127A
FVB/B6	NrasG12V	Akt
FVB/B6	Ctnnb1 S45F	Akt
FVB/B6	Ctnnb1 S45F	c-Met

Supplementary Table 5. In vivo on target analysis at SERPINA1 site by UdiTaS.

Figure	Sample	total_reads		precise_editing		small_indels(<20bp)_or_substitutions		Deletions between pegRNA and nicking sgRNA (<100bp)		Deletion (large_deletions > 100bp)		AAV_insertion	
Figure 5D and 5E		total_#	Unique_UMI#	total_#	Unique_UMI#	total_#	Unique_UMI#	total_#	Unique_UMI#	total_#	Unique_UMI#	total_#	Unique_UMI#
Figure 5D and 5E	Neg-R1	1320768	46632	96	14	377	29	54	7	9	1	0	0
Figure 5D and 5E	Neg-R2	1344784	71231	112	18	385	52	44	5	14	2	0	0

Figure 5D and 5E	Neg-R3	1127485	51684	56	5	512	38	19	3	3	1	0	0
Figure 5D and 5E	6wks-R1	1817081	77854	19854	924	4352	211	1322	71	79	13	22	3
Figure 5D and 5E	6wks-R2	1945421	81256	17526	865	2464	238	1165	62	85	11	19	3
Figure 5D and 5E	6wks-R3	1521478	65424	11548	614	3716	189	1327	55	82	10	5	1
Figure 5D and 5E	10wks-R1	1325475	63367	25513	1256	7785	419	1985	112	124	17	61	7
Figure 5D and 5E	10wks-R2	1854788	74541	27452	1274	5562	298	1428	86	75	21	32	6
Figure 5D and 5E	10wks-R3	1654876	78458	36525	2115	12235	712	2565	177	228	54	216	18

Supplementary Sequences 1. Sequences of reporter cell line used in this study

mCherry Reporter sequence for A-to-G transition :

PAM-sgRNA stop_codon

ATGGTGAGCAAGGGCGAGGAGGACAACATGGCCATCATCAAGGAGTTCATGCGCTTCAAGGTGCACATGG
AGGGCTCCGTGAACGGCCACGAGTTCGAGATCGAGGGtGAGGGtGAGGGCCGaCCCTACGAGGGCACCtA
GACCGCCAAGCTGAAGGTGACCAAGGGCGGaCCCCTGCCCTTCGCCTGGGACATCCTGTCCCCTCAGTTC
ATGTACGGCTCCAAGGCCTACGTGAAGCACCCCGCCGACATCCCCGACTACTTGAAGCTGTCCTTCCCCG
AGGGCTTCAAGTGGGAGCGCGTGATGAACTTCGAGGACGGCGGCGTGGTGACCGTGACCCAGGACTCCTC
CCTGCAGGACGGCGAGTTCATCTACAAGGTGAAGCTGCGCGGCACCAACTTCCCCTCCGACGGCCCCGTA
ATGCAGAAGAAGACCATGGGCTGGGAGGCCTCCTCCGAGCGGATGTACCCCGAGGACGGCGCCCTGAAGG
GCGAGATCAAGCAGAGGCTGAAGCTGAAGGACGGCGGCCACTACGACGCCGAGGTCAAGACCACCTACAA
GGCCAAGAAGCCCGTGCAGCTGCCCGGCGCCTACAACGTCAACATCAAGCTGGACATCACCTCCCACAAC
GAGGACTACACCATCGTGGAACAGTACGAGCGCGCCGAGGGCCGCCACTCCACCGGCGGCATGGACGAGC
TGTACAAGTAA

Traffic Light Reporter sequence for 47bp deletion:

GFP-47bp_Insertion-P2A-mCherry

ATGCCCGCCATGAAGATCGAGTGCCGCATCACCGGCACCCTGAACGGCGTGGAGTTCGAGCTGGTGGGCG
GCGGAGAGGGCACCCCcgaggggccccaaatcacctgcctcgtgggagacaaaatacccgttcCGAGCAG
GGtCGCATGACCAACAAGATGAAGAGCACCAAAGGCGCCCTGACCTTCAGCCCCTACCTGCTGAGCCACG
TGATGGGCTACGGCTTCTACCACTTCGGCACCTACCCCAGCGGCTACGAGAACCCCTTCCTGCACGCCAT
CAACAACGGCGGCTACACCAACACCCGCATCGAGAAGTACGAGGACGGCGGCGTGCTGCACGTGAGCTTC
AGCTACCGCTACGAGGCCGGCCGCGTGATCGGCGACTTCAAGGTGGTGGGCACCGGCTTCCCCGAGGACA
GCGTGATCTTCACCGACAAGATCATCCGCAGCAACGCCACCGTGGAGCACCTGCACCCCATGGGCGAcAA
CGTGCTGGTGGGCAGCTTCGCCCGCACCTTCAGCCTGCGCGACGGCGGCTACTACAGCTTCGTGGTGGAC
AGCCACATGCACTTCAAGAGCGCCATCCACCCCAGCATCCTGCAGAACGGGGGCCCCATGTTCGCCTTCC

GCCGCGTGGAGGAGCTGCACAGCAACACCGAGCTGGGCATCGTGGAGTACCAGCACGCCTTCAAGACCCC
CATCGCCTTCGCCAGATCTCGAGCTCGAggccacgaatttctcgctactcaagcaggccggcgatgtcg
aggaaaaccctggtcctGTGAGCAAGGGCGAGGAGGACAACATGGCCATCATCAAGGAGTTCATGCGCTT
CAAGGTGCACATGGAGGGCTCCGTGAACGGCCACGAGTTCGAGATCGAGGGCGAGGGCGAGGGCCGCCCC
TACGAGGGCACCCAGACCGCCAAGCTGAAGGTGACCAAGGGCGGCCCCCTGCCCTTCGCCTGGGACATCC
TGTCCCCTCAGTTCATGTACGGCTCCAAGGCCTACGTGAAGCACCCCGCCGACATCCCCGACTACTTGAA
GCTGTCCTTCCCCGAGGGCTTCAAGTGGGAGCGCGTGATGAACTTCGAGGACGGCGGCGTGGTGACCGTG
ACCCAGGACTCCTCCCTGCAGGACGGCGAGTTCATCTACAAGGTGAAGCTGCGCGGCACCAACTTCCCCT
CCGACGGCCCCGTAATGCAGAAGAAGACCATGGGCTGGGAGGCCTCCTCCGAGCGGATGTACCCCGAGGA
CGGCGCCCTGAAGGGCGAGATCAAGCAGAGGCTGAAGCTGAAGGACGGCGGCCACTACGACGCCGAGGTC
AAGACCACCTACAAGGCCAAGAAGCCCGTGCAGCTGCCCGGCGCCTACAACGTCAACATCAAGCTGGACA
TCACCTCCCACAACGAGGACTACACCATCGTGGAACAGTACGAGCGCGCCGAGGGCCGCCACTCCACCGG
CGGCATGGACGAGCTGTACAAGTAATAG

Traffic Light Reporter sequence for 39bp deletion + 18bp insertion:

GFP-39bp_Insertion-T2A-mCherry

atggtgagcaagggcgaggagctgttcaccggggtggtgcccatcctggtcgagctggacggcgacgtaa
acggccacaagttcagcgtgtccggcTTTGGCGAGACAAATCACCTGCCTCGTGGAATACGGTAAaccta
cggcaagctgaccctgaagttcatctgcaccaccggcaagctgcccgtgccctggcccaccctcgtgacc
accctgacctacggcgtgcagtgcttcagccgctaccccgaccacatgaagcagcacgacttcttcaagt
ccgccatgcccgaaggctacgtccaggagcgcaccatcttcttcaaggacgacggcaactacaagacccg
cgccgaggtgaagttcgagggcgacaccctggtgaaccgcatcgagctgaagggcatcgacttcaaggag
gacggcaacatcctggggcacaagctggagtacaactacaacagccacaacgtctatatcatggccgaca
agcagaagaacggcatcaaggtgaacttcaagatccgccacaacatcgaggacggcagcgtgcagctcgc
cgaccactaccagcagaacacccccatcggcgacggccccgtgctgctgcccgacaaccactacctgagc
acccagtccgccctgagcaaagaccccaacgagaagcgcgatcacatggtcctgctggagttcgtgaccg
ccgccgggatcactctcggcatggacgagctgtacaagtaactggatccggtgagggcagaggaagtctt
ctaacatgcggtgacgtggaggagaatccgggccctgtgagcaagggcgaggaggataactccgccatca
tcaaggagttcctgcgcttcaaggtgcacatggagggctccgtgaacggccacgagttcgagatcgaggg
cgagggcgagggccgcccctacgagggcacccagaccgccaagctgaaggtgaccaagggtggcccccctg
cccttcgcctgggacatcctgtcccctcagttcatgtacggctccaaggcctacgtgaagcaccccgccg
acatccccgactacttgaagctgtccttccccgagggcttcaagtgggagcgcgtgatgaacttcgagga
cggcggcgtggtgaccgtgacccaggactcctctctgcaggacggcgagttcatctacaaggtgaagctg
cgcggcaccaacttcccctccgacggccccgtaatgcagaagaagaccatgggctgggaggcctcctccg
agcggatgtaccccgaggacggcgccctgaagggcgagatcaagcagaggctgaagctgaaggacggcgg
ccactacgacgctgaggtcaagaccacctacaaggccaagaagcccgtgcagctgcccggcgcctacaac
gtcaacatcaagttggacatcacctcccacaacgaggactacaccatcgtggaacagtacgaacgcgccg
agggccgccactccaccggcggcatggacgagctgtacaagtga

Supplementary Sequences 2. Sequence of backbone plasmid used for pegRNA and nicking sgRNA cloning

Backbone of pegRNA used for PE2 or PE2*: U6 promoter + spCas9-sgRNA scaffold

GTGGCACTTTTCGGGGAAATGTGCGCGGAACCCCTATTTGTTTATTTTTCTAAATACATTCAAA
TATGTATCCGCTCATGAGACAATAACCCTGATAAATGCTTCAATAATATTGAAAAAGGAAGAGT
ATGAGTATTCAACATTTCCGTGTCGCCCTTATTCCCTTTTTTGCGGCATTTTGCCTTCCTGTTT
TTGCTCACCCAGAAACGCTGGTGAAAGTAAAAGATGCTGAAGATCAGTTGGGTGCACGAGTGGG
TTACATCGAACTGGATCTCAACAGCGGTAAGATCCTTGAGAGTTTTCGCCCCGAAGAACGTTTT
CCAATGATGAGCACTTTTAAAGTTCTGCTATGTGGCGCGGTATTATCCCGTATTGACGCCGGGC
AAGAGCAACTCGGTCGCCGCATACACTATTCTCAGAATGACTTGGTTGAGTACTCACCAGTCAC
AGAAAAGCATCTTACGGATGGCATGACAGTAAGAGAATTATGCAGTGCTGCCATAACCATGAGT
GATAACACTGCGGCCAACTTACTTCTGACAACGATCGGAGGACCGAAGGAGCTAACCGCTTTTT
TGCACAACATGGGGGATCATGTAACTCGCCTTGATCGTTGGGAACCGGAGCTGAATGAAGCCAT
ACCAAACGACGAGCGTGACACCACGATGCCTGTAGCAATGGCAACAACGTTGCGCAAACTATTA
ACTGGCGAACTACTTACTCTAGCTTCCCGGCAACAATTAATAGACTGGATGGAGGCGGATAAAG
TTGCAGGACCACTTCTGCGCTCGGCCCTTCCGGCTGGCTGGTTTATTGCTGATAAATCTGGAGC
CGGTGAGCGTGGGTCTCGCGGTATCATTGCAGCACTGGGGCCAGATGGTAAGCCCTCCCGTATC
GTAGTTATCTACACGACGGGGAGTCAGGCAACTATGGATGAACGAAATAGACAGATCGCTGAGA
TAGGTGCCTCACTGATTAAGCATTGGTAACTGTCAGACCAAGTTTACTCATATATACTTTAGAT
TGATTTAAAACTTCATTTTTAATTTAAAAGGATCTAGGTGAAGATCCTTTTTGATAATCTCATG
ACCAAAATCCCTTAACGTGAGTTTTCGTTCCACTGAGCGTCAGACCCCGTAGAAAAGATCAAAG
GATCTTCTTGAGATCCTTTTTTTCTGCGCGTAATCTGCTGCTTGCAAACAAAAAAACCACCGCT
ACCAGCGGTGGTTTGTTTGCCGGATCAAGAGCTACCAACTCTTTTTCCGAAGGTAACTGGCTTC
AGCAGAGCGCAGATACCAAATACTGTCCTTCTAGTGTAGCCGTAGTTAGGCCACCACTTCAAGA
ACTCTGTAGCACCGCCTACATACCTCGCTCTGCTAATCCTGTTACCAGTGGCTGCTGCCAGTGG
CGATAAGTCGTGTCTTACCGGGTTGGACTCAAGACGATAGTTACCGGATAAGGCGCAGCGGTCG
GGCTGAACGGGGGGTTCGTGCACACAGCCCAGCTTGGAGCGAACGACCTACACCGAACTGAGAT
ACCTACAGCGTGAGCTATGAGAAAGCGCCACGCTTCCCGAAGGGAGAAAGGCGGACAGGTATCC
GGTAAGCGGCAGGGTCGGAACAGGAGAGCGCACGAGGGAGCTTCCAGGGGGAAACGCCTGGTAT
CTTTATAGTCCTGTCGGGTTTCGCCACCTCTGACTTGAGCGTCGATTTTTGTGATGCTCGTCAG
GGGGGCGGAGCCTATGGAAAAACGCCAGCAACGCGGCCTTTTTACGGTTCCTGGCCTTTTGCTG
GCCTTTTGCTCACATGTTCTTTCCTGCGTTATCCCCTGATTCTGTGGATAACCGTATTACCGCC
TTTGAGTGAGCTGATACCGCTCGCCGCAGCCGAACGACCGAGCGCAGCGAGTCAGTGAGCGAGG
AAGCGGAAGAGCGCCCAATACGCAAACCGCCTCTCCCCGCGCGTTGGCCGATTCATTAATGCAG
CTGGCACGACAGGTTTCCCGACTGGAAAGCGGGCAGTGAGCGCAACGCAATTAATGTGAGTTAG
CTCACTCATTAGGCACCCCAGGCTTTACACTTTATGCTTCCGGCTCGTATGTTGTGTGGAATTG
TGAGCGGATAACAATTTCACACAGGAAACAGCTATGACCATGATTACGCCAAGCGCGCAATTAA
CCCTCACTAAAGGGAACAAAAGCTGGAGCTCCACCGCGGTGGCGGCCGCCCCCTTCACCGAGGG
CCTATTTCCCATGATTCCTTCATATTTGCATATACGATACAAGGCTGTTAGAGAGATAATTGGA
ATTAATTTGACTGTAAACACAAAGATATTAGTACAAAATACGTGACGTAGAAAGTAATAATTTC
TTGGGTAGTTTGCAGTTTTAAAATTATGTTTTAAAATGGACTATCATATGCTTACCGTAACTTG
AAAGTATTTCGATTTCTTGGCTTTATATATCTTGTGGAAAGGACGAAACACCGGTGTGCAGGTG
AGTGATCCAAACGCCCGGCGGCAACCGAGCGTTCTGAACAAATCCAGATGGAGTTCTGAGGTCA
TTACTGGATCTATCAACAGGAGTCCAAGCGAGCTCTCGAACCCCAGAGTCCCGCTCAGAAGAAC
TCGTCAAGAAGGCGATAGAAGGCGATGCGCTGCGAATCGGGAGCGGCGATACCGTAAAGCACGA
GGAAGCGGTCAGCCCATTCGCCGCCAAGCTCTTCAGCAATATCACGGGTAGCCAACGCTATGTC
CTGATAGCGGTCCGCCACACCCAGCCGGCCACAGTCGATGAATCCAGAAAAGCGGCCATTTTCC
ACCATGATATTCGGCAAGCAGGCATCGCCATGGGTCACGACGAGATCCTCGCCGTCGGGCATGC

GCGCCTTGAGCCTGGCGAACAGTTCGGCTGGCGCGAGCCCCTGATGCTCTTCGTCCAGATCATC
CTGATCGACAAGACCGGCTTCCATCCGAGTACGTGCTCGCTCGATGCGATGTTTCGCTTGGTGG
TCGAATGGGCAGGTAGCCGGATCAAGCGTATGCAGCCGCCGCATTGCATCAGCCATGATGGATA
CTTTCTCGGCAGGAGCAAGGTGAGATGACAGGAGATCCTGCCCCGGCACTTCGCCCAATAGCAG
CCAGTCCCTTCCCGCTTCAGTGACAACGTCGAGCACAGCTGCGCAAGGAACGCCCGTCGTGGCC
AGCCACGATAGCCGCGCTGCCTCGTCCTGCAGTTCATTCAGGGCACCGGACAGGTCGGTCTTGA
CAAAAAGAACCGGGCGCCCCTGCGCTGACAGCCGGAACACGGCGGCATCAGAGCAGCCGATTGT
CTGTTGTGCCCAGTCATAGCCGAATAGCCTCTCCACCCAAGCGGCCGGAGAACCTGCGTGCAAT
CCATCTTGTTCAATCATGCGAAACGATCCTCATCCTGTCTCTTGATCAGATCTTGATCCCCTGC
GCCATCAGATCCTTGGCGGCAAGAAAGCCATCCAGTTTACTTTGCAGGGCTTCCCAACCTTACC
AGAGGGCGCCCCAGCTGGCAATTCCGACGGATCAAGAACCTGCTGACGTTTTAGAGCTAGAAATAG
CAAGTTAAAATAAGGCTAGTCCGTTATCAACTTGAAAAAGTGGGACCGAGTCGGTCCTTTTTTTGAATT
CGATATCAAGCTTATCGATACCGTCGACCTCGAGGGGGGGCCCGGTACCCAATTCGCCCTATAG
TGAGTCGTATTACGCGCGCTCACTGGCCGTCGTTTTACAACGTCGTGACTGGGAAAACCCTGGC
GTTACCCAACTTAATCGCCTTGCAGCACATCCCCCTTTCGCCAGCTGGCGTAATAGCGAAGAGG
CCCGCACCGATCGCCCTTCCCAACAGTTGCGCAGCCTGAATGGCGAATGGGACGCGCCCTGTAG
CGGCGCATTAAGCGCGGCGGGTGTGGTGGTTACGCGCAGCGTGACCGCTACACTTGCCAGCGCC
CTAGCGCCCGCTCCTTTCGCTTTCTTCCCTTCCTTTCTCGCCACGTTCGCCGGCTTTCCCCGTC
AAGCTCTAAATCGGGGGCTCCCTTTAGGGTTCCGATTTAGTGCTTTACGGCACCTCGACCCCAA
AAAACTTGATTAGGGTGATGGTTCACGTAGTGGGCCATCGCCCTGATAGACGGTTTTTCGCCCT
TTGACGTTGGAGTCCACGTTCTTTAATAGTGGACTCTTGTTCCAAACTGGAACAACACTCAACC
CTATCTCGGTCTATTCTTTTGATTTATAAGGGATTTTGCCGATTTCGGCCTATTGGTTAAAAAA
TGAGCTGATTTAACAAAAATTTAACGCGAATTTTAACAAAATATTAACGCTTACAATTTAG

Backbone of Nicking sgRNA used for PE2 or PE2*: U6 promoter + spCas9-sgRNA scaffold

GTGGCACTTTTCGGGGAAATGTGCGCGGAACCCCTATTTGTTTATTTTTCTAAATACATTCAAATATGTA
TCCGCTCATGAGACAATAACCCTGATAAATGCTTCAATAATATTGAAAAAGGAAGAGTATGAGTATTCAA
CATTTCCGTGTCGCCCTTATTCCCTTTTTTGCGGCATTTTGCCTTCCTGTTTTTGCTCACCCAGAAACGC
TGGTGAAAGTAAAAGATGCTGAAGATCAGTTGGGTGCACGAGTGGGTTACATCGAACTGGATCTCAACAG
CGGTAAGATCCTTGAGAGTTTTCGCCCCGAAGAACGTTTTCCAATGATGAGCACTTTTAAAGTTCTGCTA
TGTGGCGCGGTATTATCCCGTATTGACGCCGGGCAAGAGCAACTCGGTCGCCGCATACACTATTCTCAGA
ATGACTTGGTTGAGTACTCACCAGTCACAGAAAAGCATCTTACGGATGGCATGACAGTAAGAGAATTATG
CAGTGCTGCCATAACCATGAGTGATAACACTGCGGCCAACTTACTTCTGACAACGATCGGAGGACCGAAG
GAGCTAACCGCTTTTTTGCACAACATGGGGGATCATGTAACTCGCCTTGATCGTTGGGAACCGGAGCTGA
ATGAAGCCATACCAAACGACGAGCGTGACACCACGATGCCTGTAGCAATGGCAACAACGTTGCGCAAACT
ATTAACTGGCGAACTACTTACTCTAGCTTCCCGGCAACAATTAATAGACTGGATGGAGGCGGATAAAGTT
GCAGGACCACTTCTGCGCTCGGCCCTTCCGGCTGGCTGGTTTATTGCTGATAAATCTGGAGCCGGTGAGC
GTGGGTCTCGCGGTATCATTGCAGCACTGGGGCCAGATGGTAAGCCCTCCCGTATCGTAGTTATCTACAC
GACGGGGAGTCAGGCAACTATGGATGAACGAAATAGACAGATCGCTGAGATAGGTGCCTCACTGATTAAG
CATTGGTAACTGTCAGACCAAGTTTACTCATATATACTTTAGATTGATTTAAAACTTCATTTTTAATTTA
AAAGGATCTAGGTGAAGATCCTTTTTGATAATCTCATGACCAAAATCCCTTAACGTGAGTTTTCGTTCCA
CTGAGCGTCAGACCCCGTAGAAAAGATCAAAGGATCTTCTTGAGATCCTTTTTTTCTGCGCGTAATCTGC
TGCTTGCAAACAAAAAAACCACCGCTACCAGCGGTGGTTTGTTTGCCGGATCAAGAGCTACCAACTCTTT
TTCCGAAGGTAACTGGCTTCAGCAGAGCGCAGATACCAAATACTGTCCTTCTAGTGTAGCCGTAGTTAGG
CCACCACTTCAAGAACTCTGTAGCACCGCCTACATACCTCGCTCTGCTAATCCTGTTACCAGTGGCTGCT
GCCAGTGGCGATAAGTCGTGTCTTACCGGGTTGGACTCAAGACGATAGTTACCGGATAAGGCGCAGCGGT

CGGGCTGAACGGGGGGTTCGTGCACACAGCCCAGCTTGGAGCGAACGACCTACACCGAACTGAGATACCT
ACAGCGTGAGCTATGAGAAAGCGCCACGCTTCCCGAAGGGAGAAAGGCGGACAGGTATCCGGTAAGCGGC
AGGGTCGGAACAGGAGAGCGCACGAGGGAGCTTCCAGGGGGAAACGCCTGGTATCTTTATAGTCCTGTCG
GGTTTCGCCACCTCTGACTTGAGCGTCGATTTTTGTGATGCTCGTCAGGGGGGCGGAGCCTATGGAAAAA
CGCCAGCAACGCGGCCTTTTTACGGTTCCTGGCCTTTTGCTGGCCTTTTGCTCACATGTTCTTTCCTGCG
TTATCCCCTGATTCTGTGGATAACCGTATTACCGCCTTTGAGTGAGCTGATACCGCTCGCCGCAGCCGAA
CGACCGAGCGCAGCGAGTCAGTGAGCGAGGAAGCGGAAGAGCGCCCAATACGCAAACCGCCTCTCCCCGC
GCGTTGGCCGATTCATTAATGCAGCTGGCACGACAGGTTTCCCGACTGGAAAGCGGGCAGTGAGCGCAAC
GCAATTAATGTGAGTTAGCTCACTCATTAGGCACCCCAGGCTTTACACTTTATGCTTCCGGCTCGTATGT
TGTGTGGAATTGTGAGCGGATAACAATTTCACACAGGAAACAGCTATGACCATGATTACGCCAAGCGCGC
AATTAACCCTCACTAAAGGGAACAAAAGCTGGAGCTCCACCGCGGTGGCGGCCGCCCCCTTCACCGAGGG
CCTATTTCCCATGATTCCTTCATATTTGCATATACGATACAAGGCTGTTAGAGAGATAATTGGAATTAAT
TTGACTGTAAACACAAAGATATTAGTACAAAATACGTGACGTAGAAAGTAATAATTTCTTGGGTAGTTTG
CAGTTTTAAAATTATGTTTTAAAATGGACTATCATATGCTTACCGTAACTTGAAAGTATTTCGATTTCTT
GGCTTTATATATCTTGTGGAAAGGACGAAACACCGGTGTGCAGGTGAGTGATCCAAACGCCCGGCGGCAA
CCGAGCGTTCTGAACAAATCCAGATGGAGTTCTGAGGTCATTACTGGATCTATCAACAGGAGTCCAAGCG
AGCTCTCGAACCCCAGAGTCCCGCTCAGAAGAACTCGTCAAGAAGGCGATAGAAGGCGATGCGCTGCGAA
TCGGGAGCGGCGATACCGTAAAGCACGAGGAAGCGGTCAGCCCATTCGCCGCCAAGCTCTTCAGCAATAT
CACGGGTAGCCAACGCTATGTCCTGATAGCGGTCCGCCACACCCAGCCGGCCACAGTCGATGAATCCAGA
AAAGCGGCCATTTTCCACCATGATATTCGGCAAGCAGGCATCGCCATGGGTCACGACGAGATCCTCGCCG
TCGGGCATGCGCGCCTTGAGCCTGGCGAACAGTTCGGCTGGCGCGAGCCCCTGATGCTCTTCGTCCAGAT
CATCCTGATCGACAAGACCGGCTTCCATCCGAGTACGTGCTCGCTCGATGCGATGTTTCGCTTGGTGGTC
GAATGGGCAGGTAGCCGGATCAAGCGTATGCAGCCGCCGCATTGCATCAGCCATGATGGATACTTTCTCG
GCAGGAGCAAGGTGAGATGACAGGAGATCCTGCCCCGGCACTTCGCCCAATAGCAGCCAGTCCCTTCCCG
CTTCAGTGACAACGTCGAGCACAGCTGCGCAAGGAACGCCCGTCGTGGCCAGCCACGATAGCCGCGCTGC
CTCGTCCTGCAGTTCATTCAGGGCACCGGACAGGTCGGTCTTGACAAAAAGAACCGGGCGCCCCTGCGCT
GACAGCCGGAACACGGCGGCATCAGAGCAGCCGATTGTCTGTTGTGCCCAGTCATAGCCGAATAGCCTCT
CCACCCAAGCGGCCGGAGAACCTGCGTGCAATCCATCTTGTTCAATCATGCGAAACGATCCTCATCCTGT
CTCTTGATCAGATCTTGATCCCCTGCGCCATCAGATCCTTGGCGGCAAGAAAGCCATCCAGTTTACTTTG
CAGGGCTTCCCAACCTTACCAGAGGGCGCCCCAGCTGGCAATTCCGACGGATCAAGAACCTGCTGACGTT
TTAGAGCTAGAAATAGCAAGTTAAAATAAGGCTAGTCCGTTATCAACTTGAAAAAGTGGCACCGAGTCGG
TGCTTTTTTTGAATTCGATATCAAGCTTATCGATACCGTCGACCTCGAGGGGGGGCCCGGTACCCAATTC
GCCCTATAGTGAGTCGTATTACGCGCGCTCACTGGCCGTCGTTTTACAACGTCGTGACTGGGAAAACCCT
GGCGTTACCCAACTTAATCGCCTTGCAGCACATCCCCCTTTCGCCAGCTGGCGTAATAGCGAAGAGGCCC
GCACCGATCGCCCTTCCCAACAGTTGCGCAGCCTGAATGGCGAATGGGACGCGCCCTGTAGCGGCGCATT
AAGCGCGGCGGGTGTGGTGGTTACGCGCAGCGTGACCGCTACACTTGCCAGCGCCCTAGCGCCCGCTCCT
TTCGCTTTCTTCCCTTCCTTTCTCGCCACGTTCGCCGGCTTTCCCCGTCAAGCTCTAAATCGGGGGCTCC
CTTTAGGGTTCCGATTTAGTGCTTTACGGCACCTCGACCCCAAAAAACTTGATTAGGGTGATGGTTCACG
TAGTGGGCCATCGCCCTGATAGACGGTTTTTCGCCCTTTGACGTTGGAGTCCACGTTCTTTAATAGTGGA
CTCTTGTTCCAAACTGGAACAACACTCAACCCTATCTCGGTCTATTCTTTTGATTTATAAGGATTTTGC
CGATTTCGGCCTATTGGTTAAAAAATGAGCTGATTTAACAAAAATTTAACGCGAATTTTAACAAAATATT
AACGCTTACAATTTAG

Backbone of pegRNA or Nicking sgRNA used for for SaPE2* and SaPE2*: U6 promoter + SaCas9 sgRNA scaffold

GTGGCACTTTTCGGGGAAATGTGCGCGGAACCCCTATTTGTTTATTTTTCTAAATACATTCAAATATGTA
TCCGCTCATGAGACAATAACCCTGATAAATGCTTCAATAATATTGAAAAAGGAAGAGTATGAGTATTCAA
CATTTCCGTGTCGCCCTTATTCCCTTTTTTGCGGCATTTTGCCTTCCTGTTTTTGCTCACCCAGAAACGC
TGGTGAAAGTAAAAGATGCTGAAGATCAGTTGGGTGCACGAGTGGGTTACATCGAACTGGATCTCAACAG
CGGTAAGATCCTTGAGAGTTTTCGCCCCGAAGAACGTTTTCCAATGATGAGCACTTTTAAAGTTCTGCTA
TGTGGCGCGGTATTATCCCGTATTGACGCCGGGCAAGAGCAACTCGGTCGCCGCATACACTATTCTCAGA
ATGACTTGGTTGAGTACTCACCAGTCACAGAAAAGCATCTTACGGATGGCATGACAGTAAGAGAATTATG
CAGTGCTGCCATAACCATGAGTGATAACACTGCGGCCAACTTACTTCTGACAACGATCGGAGGACCGAAG
GAGCTAACCGCTTTTTTGCACAACATGGGGGATCATGTAACTCGCCTTGATCGTTGGGAACCGGAGCTGA
ATGAAGCCATACCAAACGACGAGCGTGACACCACGATGCCTGTAGCAATGGCAACAACGTTGCGCAAACT
ATTAACTGGCGAACTACTTACTCTAGCTTCCCGGCAACAATTAATAGACTGGATGGAGGCGGATAAAGTT
GCAGGACCACTTCTGCGCTCGGCCCTTCCGGCTGGCTGGTTTATTGCTGATAAATCTGGAGCCGGTGAGC
GTGGGTCTCGCGGTATCATTGCAGCACTGGGGCCAGATGGTAAGCCCTCCCGTATCGTAGTTATCTACAC
GACGGGGAGTCAGGCAACTATGGATGAACGAAATAGACAGATCGCTGAGATAGGTGCCTCACTGATTAAG
CATTGGTAACTGTCAGACCAAGTTTACTCATATATACTTTAGATTGATTTAAAACTTCATTTTTAATTTA
AAAGGATCTAGGTGAAGATCCTTTTTGATAATCTCATGACCAAAATCCCTTAACGTGAGTTTTCGTTCCA
CTGAGCGTCAGACCCCGTAGAAAAGATCAAAGGATCTTCTTGAGATCCTTTTTTTCTGCGCGTAATCTGC
TGCTTGCAAACAAAAAAACCACCGCTACCAGCGGTGGTTTGTTTGCCGGATCAAGAGCTACCAACTCTTT
TTCCGAAGGTAACTGGCTTCAGCAGAGCGCAGATACCAAATACTGTCCTTCTAGTGTAGCCGTAGTTAGG
CCACCACTTCAAGAACTCTGTAGCACCGCCTACATACCTCGCTCTGCTAATCCTGTTACCAGTGGCTGCT
GCCAGTGGCGATAAGTCGTGTCTTACCGGGTTGGACTCAAGACGATAGTTACCGGATAAGGCGCAGCGGT
CGGGCTGAACGGGGGGTTCGTGCACACAGCCCAGCTTGGAGCGAACGACCTACACCGAACTGAGATACCT
ACAGCGTGAGCTATGAGAAAGCGCCACGCTTCCCGAAGGGAGAAAGGCGGACAGGTATCCGGTAAGCGGC
AGGGTCGGAACAGGAGAGCGCACGAGGGAGCTTCCAGGGGGAAACGCCTGGTATCTTTATAGTCCTGTCG
GGTTTCGCCACCTCTGACTTGAGCGTCGATTTTTGTGATGCTCGTCAGGGGGGCGGAGCCTATGGAAAAA
CGCCAGCAACGCGGCCTTTTTACGGTTCCTGGCCTTTTGCTGGCCTTTTGCTCACATGTTCTTTCCTGCG
TTATCCCCTGATTCTGTGGATAACCGTATTACCGCCTTTGAGTGAGCTGATACCGCTCGCCGCAGCCGAA
CGACCGAGCGCAGCGAGTCAGTGAGCGAGGAAGCGGAAGAGCGCCCAATACGCAAACCGCCTCTCCCCGC
GCGTTGGCCGATTCATTAATGCAGCTGGCACGACAGGTTTCCCGACTGGAAAGCGGGCAGTGAGCGCAAC
GCAATTAATGTGAGTTAGCTCACTCATTAGGCACCCCAGGCTTTACACTTTATGCTTCCGGCTCGTATGT
TGTGTGGAATTGTGAGCGGATAACAATTTCACACAGGAAACAGCTATGACCATGATTACGCCAAGCGCGC
AATTAACCCTCACTAAAGGGAACAAAAGCTGGAGCTCCACCGCGGTGGCGGCCGCCCCCTTCACCGAGGG
CCTATTTCCCATGATTCCTTCATATTTGCATATACGATACAAGGCTGTTAGAGAGATAATTGGAATTAAT
TTGACTGTAAACACAAAGATATTAGTACAAAATACGTGACGTAGAAAGTAATAATTTCTTGGGTAGTTTG
CAGTTTTAAAATTATGTTTTAAAATGGACTATCATATGCTTACCGTAACTTGAAAGTATTTCGATTTCTT
GGCTTTATATATCTTGTGGAAAGGACGAAACACCGGTGTGCAGGTGAGTGATCCAAACGCCCGGCGGCAA
CCGAGCGTTCTGAACAAATCCAGATGGAGTTCTGAGGTCATTACTGGATCTATCAACAGGAGTCCAAGCG
AGCTCTCGAACCCCAGAGTCCCGCTCAGAAGAACTCGTCAAGAAGGCGATAGAAGGCGATGCGCTGCGAA
TCGGGAGCGGCGATACCGTAAAGCACGAGGAAGCGGTCAGCCCATTCGCCGCCAAGCTCTTCAGCAATAT
CACGGGTAGCCAACGCTATGTCCTGATAGCGGTCCGCCACACCCAGCCGGCCACAGTCGATGAATCCAGA
AAAGCGGCCATTTTCCACCATGATATTCGGCAAGCAGGCATCGCCATGGGTCACGACGAGATCCTCGCCG
TCGGGCATGCGCGCCTTGAGCCTGGCGAACAGTTCGGCTGGCGCGAGCCCCTGATGCTCTTCGTCCAGAT
CATCCTGATCGACAAGACCGGCTTCCATCCGAGTACGTGCTCGCTCGATGCGATGTTTCGCTTGGTGGTC
GAATGGGCAGGTAGCCGGATCAAGCGTATGCAGCCGCCGCATTGCATCAGCCATGATGGATACTTTCTCG
GCAGGAGCAAGGTGAGATGACAGGAGATCCTGCCCCGGCACTTCGCCCAATAGCAGCCAGTCCCTTCCCG
CTTCAGTGACAACGTCGAGCACAGCTGCGCAAGGAACGCCCGTCGTGGCCAGCCACGATAGCCGCGCTGC

CTCGTCCTGCAGTTCATTCAGGGCACCGGACAGGTCGGTCTTGACAAAAAGAACCGGGCGCCCCTGCGCT
GACAGCCGGAACACGGCGGCATCAGAGCAGCCGATTGTCTGTTGTGCCCAGTCATAGCCGAATAGCCTCT
CCACCCAAGCGGCCGGAGAACCTGCGTGCAATCCATCTTGTTCAATCATGCGAAACGATCCTCATCCTGT
CTCTTGATCAGATCTTGATCCCCTGCGCCATCAGATCCTTGGCGGCAAGAAAGCCATCCAGTTTACTTTG
CAGGGCTTCCCAACCTTACCAGAGGGCGCCCCAGCTGGCAATTCCGACGGATCAgctagcAGAACCTGCT
GACGTTTTAGTACTCTGGAAACAGAATCTACTAAAACAAGGCAAAATGCCGTGTTTATCTCGTCAACTTG
TTGGCGAGATTTTTTTGAATTCGATATCAAGCTTATCGATACCGTCGACCTCGAGGGGGGGCCCGGTACC
CAATTCGCCCTATAGTGAGTCGTATTACGCGCGCTCACTGGCCGTCGTTTTACAACGTCGTGACTGGGAA
AACCCTGGCGTTACCCAACTTAATCGCCTTGCAGCACATCCCCCTTTCGCCAGCTGGCGTAATAGCGAAG
AGGCCCGCACCGATCGCCCTTCCCAACAGTTGCGCAGCCTGAATGGCGAATGGGACGCGCCCTGTAGCGG
CGCATTAAGCGCGGCGGGTGTGGTGGTTACGCGCAGCGTGACCGCTACACTTGCCAGCGCCCTAGCGCCC
GCTCCTTTCGCTTTCTTCCCTTCCTTTCTCGCCACGTTCGCCGGCTTTCCCCGTCAAGCTCTAAATCGGG
GGCTCCCTTTAGGGTTCCGATTTAGTGCTTTACGGCACCTCGACCCCAAAAAACTTGATTAGGGTGATGG
TTCACGTAGTGGGCCATCGCCCTGATAGACGGTTTTTCGCCCTTTGACGTTGGAGTCCACGTTCTTTAAT
AGTGGACTCTTGTTCCAAACTGGAACAACACTCAACCCTATCTCGGTCTATTCTTTTGATTTATAAGGGA
TTTTGCCGATTTCGGCCTATTGGTTAAAAAATGAGCTGATTTAACAAAAATTTAACGCGAATTTTAACAA
AATATTAACGCTTACAATTTAG

PegRNA used for for SaPE2* : U6 promoter-sgRNA-Scaffold-RT_template-PBS (CCR5 pegRNA as an example)

GTGGCACTTTTCGGGGAAATGTGCGCGGAACCCCTATTTGTTTATTTTTCTAAATACATTCAAATATGTA
TCCGCTCATGAGACAATAACCCTGATAAATGCTTCAATAATATTGAAAAAGGAAGAGTATGAGTATTCAA
CATTTCCGTGTCGCCCTTATTCCCTTTTTTGCGGCATTTTGCCTTCCTGTTTTTGCTCACCCAGAAACGC
TGGTGAAAGTAAAAGATGCTGAAGATCAGTTGGGTGCACGAGTGGGTTACATCGAACTGGATCTCAACAG
CGGTAAGATCCTTGAGAGTTTTCGCCCCGAAGAACGTTTTCCAATGATGAGCACTTTTAAAGTTCTGCTA
TGTGGCGCGGTATTATCCCGTATTGACGCCGGGCAAGAGCAACTCGGTCGCCGCATACACTATTCTCAGA
ATGACTTGGTTGAGTACTCACCAGTCACAGAAAAGCATCTTACGGATGGCATGACAGTAAGAGAATTATG
CAGTGCTGCCATAACCATGAGTGATAACACTGCGGCCAACTTACTTCTGACAACGATCGGAGGACCGAAG
GAGCTAACCGCTTTTTTGCACAACATGGGGGATCATGTAACTCGCCTTGATCGTTGGGAACCGGAGCTGA
ATGAAGCCATACCAAACGACGAGCGTGACACCACGATGCCTGTAGCAATGGCAACAACGTTGCGCAAACT
ATTAACTGGCGAACTACTTACTCTAGCTTCCCGGCAACAATTAATAGACTGGATGGAGGCGGATAAAGTT
GCAGGACCACTTCTGCGCTCGGCCCTTCCGGCTGGCTGGTTTATTGCTGATAAATCTGGAGCCGGTGAGC
GTGGGTCTCGCGGTATCATTGCAGCACTGGGGCCAGATGGTAAGCCCTCCCGTATCGTAGTTATCTACAC
GACGGGGAGTCAGGCAACTATGGATGAACGAAATAGACAGATCGCTGAGATAGGTGCCTCACTGATTAAG
CATTGGTAACTGTCAGACCAAGTTTACTCATATATACTTTAGATTGATTTAAAACTTCATTTTTAATTTA
AAAGGATCTAGGTGAAGATCCTTTTTGATAATCTCATGACCAAAATCCCTTAACGTGAGTTTTCGTTCCA
CTGAGCGTCAGACCCCGTAGAAAAGATCAAAGGATCTTCTTGAGATCCTTTTTTTCTGCGCGTAATCTGC
TGCTTGCAAACAAAAAAACCACCGCTACCAGCGGTGGTTTGTTTGCCGGATCAAGAGCTACCAACTCTTT
TTCCGAAGGTAACTGGCTTCAGCAGAGCGCAGATACCAAATACTGTCCTTCTAGTGTAGCCGTAGTTAGG
CCACCACTTCAAGAACTCTGTAGCACCGCCTACATACCTCGCTCTGCTAATCCTGTTACCAGTGGCTGCT
GCCAGTGGCGATAAGTCGTGTCTTACCGGGTTGGACTCAAGACGATAGTTACCGGATAAGGCGCAGCGGT
CGGGCTGAACGGGGGGTTCGTGCACACAGCCCAGCTTGGAGCGAACGACCTACACCGAACTGAGATACCT
ACAGCGTGAGCTATGAGAAAGCGCCACGCTTCCCGAAGGGAGAAAGGCGGACAGGTATCCGGTAAGCGGC
AGGGTCGGAACAGGAGAGCGCACGAGGGAGCTTCCAGGGGGAAACGCCTGGTATCTTTATAGTCCTGTCG
GGTTTCGCCACCTCTGACTTGAGCGTCGATTTTTGTGATGCTCGTCAGGGGGGCGGAGCCTATGGAAAAA

CGCCAGCAACGCGGCCTTTTTACGGTTCCTGGCCTTTTGCTGGCCTTTTGCTCACATGTTCTTTCCTGCG
TTATCCCCTGATTCTGTGGATAACCGTATTACCGCCTTTGAGTGAGCTGATACCGCTCGCCGCAGCCGAA
CGACCGAGCGCAGCGAGTCAGTGAGCGAGGAAGCGGAAGAGCGCCCAATACGCAAACCGCCTCTCCCCGC
GCGTTGGCCGATTCATTAATGCAGCTGGCACGACAGGTTTCCCGACTGGAAAGCGGGCAGTGAGCGCAAC
GCAATTAATGTGAGTTAGCTCACTCATTAGGCACCCCAGGCTTTACACTTTATGCTTCCGGCTCGTATGT
TGTGTGGAATTGTGAGCGGATAACAATTTCACACAGGAAACAGCTATGACCATGATTACGCCAAGCGCGC
AATTAACCCTCACTAAAGGGAACAAAAGCTGGAGCTCCACCGCGGTGGCGGCCGCCCCCTTCACCGAGGG
CCTATTTCCCATGATTCCTTCATATTTGCATATACGATACAAGGCTGTTAGAGAGATAATTGGAATTAAT
TTGACTGTAAACACAAAGATATTAGTACAAAATACGTGACGTAGAAAGTAATAATTTCTTGGGTAGTTTG
CAGTTTTAAAATTATGTTTTAAAATGGACTATCATATGCTTACCGTAACTTGAAAGTATTTCGATTTCTT
GGCTTTATATATCTTGTGGAAGGACGAAACACCGAAGATGACTATCTTTAATGTCTGTTTTAGTACTCTGGAA
ACAGAATCTACTAAAACAAGGCAAAATGCCGTGTTTATCTCGTCAACTTGTTGGCGAGAAGCTCTCATTTTCCATACATT
AAAGATAGTCATCTTTTTTTGAATTCGATATCAAGCTTATCGATACCGTCGACCTCGAGGGGGGGCCCGG
TACCCAATTCGCCCTATAGTGAGTCGTATTACGCGCGCTCACTGGCCGTCGTTTTACAACGTCGTGACTG
GGAAAACCCTGGCGTTACCCAACTTAATCGCCTTGCAGCACATCCCCCTTTCGCCAGCTGGCGTAATAGC
GAAGAGGCCCGCACCGATCGCCCTTCCCAACAGTTGCGCAGCCTGAATGGCGAATGGACGCGCCCTGTA
GCGGCGCATTAAGCGCGGCGGGTGTGGTGGTTACGCGCAGCGTGACCGCTACACTTGCCAGCGCCCTAGC
GCCCGCTCCTTTCGCTTTCTTCCCTTCCTTTCTCGCCACGTTCGCCGGCTTTCCCCGTCAAGCTCTAAAT
CGGGGGCTCCCTTTAGGGTTCCGATTTAGTGCTTTACGGCACCTCGACCCCAAAAAACTTGATTAGGGTG
ATGGTTCACGTAGTGGGCCATCGCCCTGATAGACGGTTTTTCGCCCTTTGACGTTGGAGTCCACGTTCTT
TAATAGTGGACTCTTGTTCCAAACTGGAACAACACTCAACCCTATCTCGGTCTATTCTTTTGATTTATAA
GGGATTTTGCCGATTTCGGCCTATTGGTTAAAAAATGAGCTGATTTAACAAAAATTTAACGCGAATTTTA
ACAAAATATTAACGCTTACAATTTAG

Supplementary Sequences 3. Protein Sequences of prime editors

PE2: BPSV40_NLS-SpCas9H840A-linker-M-MLV_reverse_transcriptase-BPSV40

KRTADGSEFESPKKKRKVDKKYSIGLDIGTNSVGWAVITDEYKVPSKKFKVLGNTDRHSIKKNLIGALLF
DSGETAEATRLKRTARRRYTRRKNRICYLQEIFSNEMAKVDDSFFHRLEESFLVEEDKKHERHPIFGNIV
DEVAYHEKYPTIYHLRKKLVDSTDKADLRLIYLALAHMIKFRGHFLIEGDLNPDNSDVDKLFIQLVQTYN
QLFEENPINASGVDAKAILSARLSKSRRLENLIAQLPGEKKNGLFGNLIALSLGLTPNFKSNFDLAEDAK
LQLSKDTYDDDLDNLLAQIGDQYADLFLAAKNLSDAILLSDILRVNTEITKAPLSASMIKRYDEHHQDLT
LLKALVRQQLPEKYKEIFFDQSKNGYAGYIDGGASQEEFYKFIKPILEKMDGTEELLVKLNREDLLRKQR
TFDNGSIPHQIHLGELHAILRRQEDFYPFLKDNREKIEKILTFRIPYYVGPLARGNSRFAWMTRKSEETI
TPWNFEEVVDKGASAQSFIERMTNFDKNLPNEKVLPKHSLLYEYFTVYNELTKVKYVTEGMRKPAFLSGE
QKKAIVDLLFKTNRKVTVKQLKEDYFKKIECFDSVEISGVEDRFNASLGTYHDLLKIIKDKDFLDNEENE
DILEDIVLTLTLFEDREMIEERLKTYAHLFDDKVMKQLKRRRYTGWGRLSRKLINGIRDKQSGKTILDFL
KSDGFANRNFMQLIHDDSLTFKEDIQKAQVSGQGDSLHEHIANLAGSPAIKKGILQTVKVVDELVKVMGR
HKPENIVIEMARENQTTQKGQKNSRERMKRIEEGIKELGSQILKEHPVENTQLQNEKLYLYYLQNGRDMY
VDQELDINRLSDYDVDAIVPQSFLKDDSIDNKVLTRSDKNRGKSDNVPSEEVVKKMKNYWRQLLNAKLIT
QRKFDNLTKAERGGLSELDKAGFIKRQLVETRQITKHVAQILDSRMNTKYDENDKLIREVKVITLKSKLV
SDFRKDFQFYKVREINNYHHAHDAYLNAVVGTALIKKYPKLESEFVYGDYKVYDVRKMIAKSEQEIGKAT
AKYFFYSNIMNFFKTEITLANGEIRKRPLIETNGETGEIVWDKGRDFATVRKVLSMPQVNIVKKTEVQTG
GFSKESILPKRNSDKLIARKKDWDPKKYGGFDSPTVAYSVLVVAKVEKGKSKKLKSVKELLGITIMERSS
FEKNPIDFLEAKGYKEVKKDLIIKLPKYSLFELENGRKRMLASAGELQKGNELALPSKYVNFLYLASHYE

KLKGSPEDNEQKQLFVEQHKHYLDEIIEQISEFSKRVILADANLDKVLSAYNKHRDKPIREQAENIIHLF
TLTNLGAPAAFKYFDTTIDRKRYTSTKEVLDATLIHQSITGLYETRIDLSQLGGDSGGSSGGSSGSETPG
TSESATPESSGGSSGGSSTLNIEDEYRLHETSKEPDVSLGSTWLSDFPQAWAETGGMGLAVRQAPLIIPL
KATSTPVSIKQYPMSQEARLGIKPHIQRLLDQGILVPCQSPWNTPLLPVKKPGTNDYRPVQDLREVNKRV
EDIHPTVPNPYNLLSGLPPSHQWYTVLDLKDAFFCLRLHPTSQPLFAFEWRDPEMGISGQLTWTRLPQGF
KNSPTLFNEALHRDLADFRIQHPDLILLQYVDDLLLAATSELDCQQGTRALLQTLGNLGYRASAKKAQIC
QKQVKYLGYLLKEGQRWLTEARKETVMGQPTPKTPRQLREFLGKAGFCRLFIPGFAEMAAPLYPLTKPGT
LFNWGPDQQKAYQEIKQALLTAPALGLPDLTKPFELFVDEKQGYAKGVLTQKLGPWRRPVAYLSKKLDPV
AAGWPPCLRMVAAIAVLTKDAGKLTMGQPLVILAPHAVEALVKQPPDRWLSNARMTHYQALLLDTDRVQF
GPVVALNPATLLPLPEEGLQHNCLDILAEAHGTRPDLTDQPLPDADHTWYTDGSSLLQEGQRKAGAAVTT
ETEVIWAKALPAGTSAQRAELIALTQALKMAEGKKLNVYTDSRYAFATAHIHGEIYRRRGWLTSEGKEIK
NKDEILALLKALFLPKRLSIIHCPGHQKGHSAEARGNRMADQAARKAAITETPDTSTLLIENSSPSGGSK
RTADGSEFEPKKKRKV

PE2*: Cmyc_NLS-BPSV40_NLS-SpCas9H840A-linker-M-MLV_reverse_transcriptase-vBPSV40_NLS-SV40

PAAKRVKLDGGKRTADGSEFESPKKKRKVDKKYSIGLDIGTNSVGWAVITDEYKVPSKKFKVLGNTDRHS
IKKNLIGALLFDSGETAEATRLKRTARRRYTRRKNRICYLQEIFSNEMAKVDDSFFHRLEESFLVEEDKK
HERHPIFGNIVDEVAYHEKYPTIYHLRKKLVDSTDKADLRLIYLALAHMIKFRGHFLIEGDLNPDNSDVD
KLFIQLVQTYNQLFEENPINASGVDAKAILSARLSKSRRLENLIAQLPGEKKNGLFGNLIALSLGLTPNF
KSNFDLAEDAKLQLSKDTYDDDLDNLLAQIGDQYADLFLAAKNLSDAILLSDILRVNTEITKAPLSASMI
KRYDEHHQDLTLLKALVRQQLPEKYKEIFFDQSKNGYAGYIDGGASQEEFYKFIKPILEKMDGTEELLVK
LNREDLLRKQRTFDNGSIPHQIHLGELHAILRRQEDFYPFLKDNREKIEKILTFRIPYYVGPLARGNSRF
AWMTRKSEETITPWNFEEVVDKGASAQSFIERMTNFDKNLPNEKVLPKHSLLYEYFTVYNELTKVKYVTE
GMRKPAFLSGEQKKAIVDLLFKTNRKVTVKQLKEDYFKKIECFDSVEISGVEDRFNASLGTYHDLLKIIK
DKDFLDNEENEDILEDIVLTLTLFEDREMIEERLKTYAHLFDDKVMKQLKRRRYTGWGRLSRKLINGIRD
KQSGKTILDFLKSDGFANRNFMQLIHDDSLTFKEDIQKAQVSGQGDSLHEHIANLAGSPAIKKGILQTVK
VVDELVKVMGRHKPENIVIEMARENQTTQKGQKNSRERMKRIEEGIKELGSQILKEHPVENTQLQNEKLY
LYYLQNGRDMYVDQELDINRLSDYDVDAIVPQSFLKDDSIDNKVLTRSDKNRGKSDNVPSEEVVKKMKNY
WRQLLNAKLITQRKFDNLTKAERGGLSELDKAGFIKRQLVETRQITKHVAQILDSRMNTKYDENDKLIRE
VKVITLKSKLVSDFRKDFQFYKVREINNYHHAHDAYLNAVVGTALIKKYPKLESEFVYGDYKVYDVRKMI
AKSEQEIGKATAKYFFYSNIMNFFKTEITLANGEIRKRPLIETNGETGEIVWDKGRDFATVRKVLSMPQV
NIVKKTEVQTGGFSKESILPKRNSDKLIARKKDWDPKKYGGFDSPTVAYSVLVVAKVEKGKSKKLKSVKE
LLGITIMERSSFEKNPIDFLEAKGYKEVKKDLIIKLPKYSLFELENGRKRMLASAGELQKGNELALPSKY
VNFLYLASHYEKLKGSPEDNEQKQLFVEQHKHYLDEIIEQISEFSKRVILADANLDKVLSAYNKHRDKPI
REQAENIIHLFTLTNLGAPAAFKYFDTTIDRKRYTSTKEVLDATLIHQSITGLYETRIDLSQLGGDSGGS
SGGSSGSETPGTSESATPESSGGSSGGSSTLNIEDEYRLHETSKEPDVSLGSTWLSDFPQAWAETGGMGL
AVRQAPLIIPLKATSTPVSIKQYPMSQEARLGIKPHIQRLLDQGILVPCQSPWNTPLLPVKKPGTNDYRP
VQDLREVNKRVEDIHPTVPNPYNLLSGLPPSHQWYTVLDLKDAFFCLRLHPTSQPLFAFEWRDPEMGISG
QLTWTRLPQGFKNSPTLFNEALHRDLADFRIQHPDLILLQYVDDLLLAATSELDCQQGTRALLQTLGNLG
YRASAKKAQICQKQVKYLGYLLKEGQRWLTEARKETVMGQPTPKTPRQLREFLGKAGFCRLFIPGFAEMA
APLYPLTKPGTLFNWGPDQQKAYQEIKQALLTAPALGLPDLTKPFELFVDEKQGYAKGVLTQKLGPWRRP
VAYLSKKLDPVAAGWPPCLRMVAAIAVLTKDAGKLTMGQPLVILAPHAVEALVKQPPDRWLSNARMTHYQ
ALLLDTDRVQFGPVVALNPATLLPLPEEGLQHNCLDILAEAHGTRPDLTDQPLPDADHTWYTDGSSLLQE
GQRKAGAAVTTETEVIWAKALPAGTSAQRAELIALTQALKMAEGKKLNVYTDSRYAFATAHIHGEIYRRR

GWLTSEGKEIKNKDEILALLKALFLPKRLSIIHCPGHQKGHSAEARGNRMADQAARKAAITETPDTSTLL
IENSSPSGGSKRTADGSEKRTADSQHSTPPKTKRKVEFEPKKKRKV

SaPE2*: Cmyc_NLS-BPSV40_NLS-SaCas9N580A-linker-M-MLV_reverse_transcriptase-vBPSV40_NLS-SV40

PAAKRVKLDGGKRTADGSEFESPKKKRKVGIHGVPAAKRNYILGLDIGITSVGYGIIDYETRDVIDAGVR
LFKEANVENNEGRRSKRGARRLKRRRRHRIQRVKKLLFDYNLLTDHSELSGINPYEARVKGLSQKLSEEE
FSAALLHLAKRRGVHNVNEVEEDTGNELSTKEQISRNSKALEEKYVAELQLERLKKDGEVRGSINRFKTS
DYVKEAKQLLKVQKAYHQLDQSFIDTYIDLLETRRTYYEGPGEGSPFGWKDIKEWYEMLMGHCTYFPEEL
RSVKYAYNADLYNALNDLNNLVITRDENEKLEYYEKFQIIENVFKQKKKPTLKQIAKEILVNEEDIKGYR
VTSTGKPEFTNLKVYHDIKDITARKEIIENAELLDQIAKILTIYQSSEDIQEELTNLNSELTQEEIEQIS
NLKGYTGTHNLSLKAINLILDELWHTNDNQIAIFNRLKLVPKKVDLSQQKEIPTTLVDDFILSPVVKRSF
IQSIKVINAIIKKYGLPNDIIIELAREKNSKDAQKMINEMQKRNRQTNERIEEIIRTTGKENAKYLIEKI
KLHDMQEGKCLYSLEAIPLEDLLNNPFNYEVDHIIPRSVSFDNSFNNKVLVKQEEASKKGNRTPFQYLSS
SDSKISYETFKKHILNLAKGKGRISKTKKEYLLEERDINRFSVQKDFINRNLVDTRYATRGLMNLLRSYF
RVNNLDVKVKSINGGFTSFLRRKWKFKKERNKGYKHHAEDALIIANADFIFKEWKKLDKAKKVMENQMFE
EKQAESMPEIETEQEYKEIFITPHQIKHIKDFKDYKYSHRVDKKPNRELINDTLYSTRKDDKGNTLIVNN
LNGLYDKDNDKLKKLINKSPEKLLMYHHDPQTYQKLKLIMEQYGDEKNPLYKYYEETGNYLTKYSKKDNG
PVIKKIKYYGNKLNAHLDITDDYPNSRNKVVKLSLKPYRFDVYLDNGVYKFVTVKNLDVIKKENYYEVNS
KCYEEAKKLKKISNQAEFIASFYNNDLIKINGELYRVIGVNNDLLNRIEVNMIDITYREYLENMNDKRPP
RIIKTIASKTQSIKKYSTDILGNLYEVKSKKHPQIIKKGSGGSSGGSSGSETPGTSESATPESSGGSSGG
SSTLNIEDEYRLHETSKEPDVSLGSTWLSDFPQAWAETGGMGLAVRQAPLIIPLKATSTPVSIKQYPMSQ
EARLGIKPHIQRLLDQGILVPCQSPWNTPLLPVKKPGTNDYRPVQDLREVNKRVEDIHPTVPNPYNLLSG
LPPSHQWYTVLDLKDAFFCLRLHPTSQPLFAFEWRDPEMGISGQLTWTRLPQGFKNSPTLFNEALHRDLA
DFRIQHPDLILLQYVDDLLLAATSELDCQQGTRALLQTLGNLGYRASAKKAQICQKQVKYLGYLLKEGQR
WLTEARKETVMGQPTPKTPRQLREFLGKAGFCRLFIPGFAEMAAPLYPLTKPGTLFNWGPDQQKAYQEIK
QALLTAPALGLPDLTKPFELFVDEKQGYAKGVLTQKLGPWRRPVAYLSKKLDPVAAGWPPCLRMVAAIAV
LTKDAGKLTMGQPLVILAPHAVEALVKQPPDRWLSNARMTHYQALLLDTDRVQFGPVVALNPATLLPLPE
EGLQHNCLDILAEAHGTRPDLTDQPLPDADHTWYTDGSSLLQEGQRKAGAAVTTETEVIWAKALPAGTSA
QRAELIALTQALKMAEGKKLNVYTDSRYAFATAHIHGEIYRRRGWLTSEGKEIKNKDEILALLKALFLPK
RLSIIHCPGHQKGHSAEARGNRMADQAARKAAITETPDTSTLLIENSSPSGGSKRTADGSEKRTADSQHS
TPPKTKRKVEFEPKKKRKV

Sa^{KKH}PE*: Cmyc_NLS-BPSV40_NLS-SaCas9^{KKH}N580A-linker-M-MLV_reverse_transcriptase-vBPSV40_NLS-SV40

PAAKRVKLDGGKRTADGSEFESPKKKRKVGIHGVPAAKRNYILGLDIGITSVGYGIIDYETRDVIDAGVR
LFKEANVENNEGRRSKRGARRLKRRRRHRIQRVKKLLFDYNLLTDHSELSGINPYEARVKGLSQKLSEEE
FSAALLHLAKRRGVHNVNEVEEDTGNELSTKEQISRNSKALEEKYVAELQLERLKKDGEVRGSINRFKTS
DYVKEAKQLLKVQKAYHQLDQSFIDTYIDLLETRRTYYEGPGEGSPFGWKDIKEWYEMLMGHCTYFPEEL
RSVKYAYNADLYNALNDLNNLVITRDENEKLEYYEKFQIIENVFKQKKKPTLKQIAKEILVNEEDIKGYR
VTSTGKPEFTNLKVYHDIKDITARKEIIENAELLDQIAKILTIYQSSEDIQEELTNLNSELTQEEIEQIS
NLKGYTGTHNLSLKAINLILDELWHTNDNQIAIFNRLKLVPKKVDLSQQKEIPTTLVDDFILSPVVKRSF
IQSIKVINAIIKKYGLPNDIIIELAREKNSKDAQKMINEMQKRNRQTNERIEEIIRTTGKENAKYLIEKI
KLHDMQEGKCLYSLEAIPLEDLLNNPFNYEVDHIIPRSVSFDNSFNNKVLVKQEEASKKGNRTPFQYLSS

SDSKISYETFKKHILNLAKGKGRISKTKKEYLLEERDINRFSVQKDFINRNLVDTRYATRGLMNLLRSYF
RVNNLDVKVKSINGGFTSFLRRKWKFKKERNKGYKHHAEDALIIANADFIFKEWKKLDKAKKVMENQMFE
EKQAESMPEIETEQEYKEIFITPHQIKHIKDFKDYKYSHRVDKKPNRKLINDTLYSTRKDDKGNTLIVNN
LNGLYDKDNDKLKKLINKSPEKLLMYHHDPQTYQKLKLIMEQYGDEKNPLYKYYEETGNYLTKYSKKDNG
PVIKKIKYYGNKLNAHLDITDDYPNSRNKVVKLSLKPYRFDVYLDNGVYKFVTVKNLDVIKKENYYEVNS
KCYEEAKKLKKISNQAEFIASFYKNDLIKINGELYRVIGVNNDLLNRIEVNMIDITYREYLENMNDKRPP
HIIKTIASKTQSIKKYSTDILGNLYEVKSKKHPQIIKKGSGGSSGGSSGSETPGTSESATPESSGGSSGG
SSTLNIEDEYRLHETSKEPDVSLGSTWLSDFPQAWAETGGMGLAVRQAPLIIPLKATSTPVSIKQYPMSQ
EARLGIKPHIQRLLDQGILVPCQSPWNTPLLPVKKPGTNDYRPVQDLREVNKRVEDIHPTVPNPYNLLSG
LPPSHQWYTVLDLKDAFFCLRLHPTSQPLFAFEWRDPEMGISGQLTWTRLPQGFKNSPTLFNEALHRDLA
DFRIQHPDLILLQYVDDLLLAATSELDCQQGTRALLQTLGNLGYRASAKKAQICQKQVKYLGYLLKEGQR
WLTEARKETVMGQPTPKTPRQLREFLGKAGFCRLFIPGFAEMAAPLYPLTKPGTLFNWGPDQQKAYQEIK
QALLTAPALGLPDLTKPFELFVDEKQGYAKGVLTQKLGPWRRPVAYLSKKLDPVAAGWPPCLRMVAAIAV
LTKDAGKLTMGQPLVILAPHAVEALVKQPPDRWLSNARMTHYQALLLDTDRVQFGPVVALNPATLLPLPE
EGLQHNCLDILAEAHGTRPDLTDQPLPDADHTWYTDGSSLLQEGQRKAGAAVTTETEVIWAKALPAGTSA
QRAELIALTQALKMAEGKKLNVYTDSRYAFATAHIHGEIYRRRGWLTSEGKEIKNKDEILALLKALFLPK
RLSIIHCPGHQKGHSAEARGNRMADQAARKAAITETPDTSTLLIENSSPSGGSKRTADGSEKRTADSQHS
TPPKTKRKVEFEPKKKRKV

pU6-Ctnnb1_pegRNA_S45F: U6 promoter + Spacer + sgRNA scaffold + RT +PBS

GAGGGCCTATTTCCCATGATTCCTTCATATTTGCATATACGATACAAGGCTGTTAGAGAGAT
AATTGGAATTAATTTGACTGTAAACACAAAGATATTAGTACAAAATACGTGACGTAGAAAGT
AATAATTTCTTGGGTAGTTTGCAGTTTTAAAATTATGTTTTAAAATGGACTATCATATGCTTA
CCGTAACTTGAAAGTATTTCGATTTCTTGGCTTTATATATCTTGTGGAAAGGACGAAACACC
GAGGGTTGCCCTTGCCACTCAgttttagagctagaaatagcaagttaaaataaggctagtccgttatcaacttgaaaaa
gtgggaccgagtcggtccGCTCCTTTCCTGAGTGGCAAGGGCAATTTTTTT

U6-pegAAT-U6-Nicking-U1A-Cmyc_NLS-SpCas9(N)-Nter-Npu-intein

cctgcaggcagctgcgcgctcgctcgctcactgaggccgcccgggcaaagcccgggcgtcgggcgacctt
tggtcgcccggcctcagtgagcgagcgagcgcgcagagagggagtggccaactccatcactaggggttcc
tgcggcctctagaggtaccGAGGGCCTATTTCCCATGATTCCTTCATATTTGCATATACGATACAAGGCT
GTTAGAGAGATAATTGGAATTAATTTGACTGTAAACACAAAGATATTAGTACAAAATACGTGACGTAGAA
AGTAATAATTTCTTGGGTAGTTTGCAGTTTTAAAATTATGTTTTAAAATGGACTATCATATGCTTACCGT
AACTTGAAAGTATTTCGATTTCTTGGCTTTATATATCTTGTGGAAAGGACGAAACACCGtcccctccagg
ccgtgcatagttttagagctagaaatagcaagttaaaataaggctagtccgttatcaacttgaaaaagtg
ggaccgagtcggtcctctcgtcgatggtcagcacagcTttatgcacggcctggagTTTTTTTGAATTCGA
TATCAAGCTTATCGATACCGTCGACCTCGgctgccgctggaggtgctcaaagagatggaGAGGGCCTATT
TCCCATGATTCCTTCATATTTGCATATACGATACAAGGCTGTTAGAGAGATAATTGGAATTAATTTGACT
GTAAACACAAAGATATTAGTACAAAATACGTGACGTAGAAAGTAATAATTTCTTGGGTAGTTTGCAGTTT
TAAAATTATGTTTTAAAATGGACTATCATATGCTTACCGTAACTTGAAAGTATTTCGATTTCTTGGCTTT
ATATATCTTGTGGAAAGGACGAAACACCGTTCAATCATTAAGAAGACAAgttttagagctagaaatagca
agttaaaataaggctagtccgttatcaacttgaaaaagtgggaccgagtcggtgcTTTTTTTGAATTCGA
TATCAAGCTTATCGATACCGTCgctagcccgggtcgactagtgatcagtgtgagggagtgtaaagctggt
ttaaacgttgttgtttggggttgaattactcttctagaccgcggcgcgcctccatggatatcaagcttat

ggaggcggtactatgtagatgagaattcaggagcaaactgggaaaagcaactgcttccaaatatttgtga
tttttacagtgtagttttggaaaaactcttagcctaccaattcttctaagtgttttaaaatgtgggagcc
agtacacatgaagttatagagtgttttaatgaggcttaaatatttaccgtaactatgaaatgctacgcat
atcatgctgttcaggctccgtggccacgcaactcatactaccggtGCCACCATGgctagcCCCGCCGCCA
AGCGCGTGAAGCTGGACGACAAGAAGTACAGCATCGGCCTGGACATCGGCACCAACTCTGTGGGCTGGGC
CGTGATCACCGACGAGTACAAGGTGCCCAGCAAGAAATTCAAGGTGCTGGGCAACACCGACCGGCACAGC
ATCAAGAAGAACCTGATCGGAGCCCTGCTGTTCGACAGCGGCGAAACAGCCGAGGCCACCCGGCTGAAGA
GAACCGCCAGAAGAAGATACACCAGACGGAAGAACCGGATCTGCTATCTGCAAGAGATCTTCAGCAACGA
GATGGCCAAGGTGGACGACAGCTTCTTCCACAGACTGGAAGAGTCCTTCCTGGTGGAAGAGGATAAGAAG
CACGAGCGGCACCCCATCTTCGGCAACATCGTGGACGAGGTGGCCTACCACGAGAAGTACCCCACCATCT
ACCACCTGAGAAAGAAACTGGTGGACAGCACCGACAAGGCCGACCTGCGGCTGATCTATCTGGCCCTGGC
CCACATGATCAAGTTCCGGGGCCACTTCCTGATCGAGGGCGACCTGAACCCCGACAACAGCGACGTGGAC
AAGCTGTTCATCCAGCTGGTGCAGACCTACAACCAGCTGTTCGAGGAAAACCCCATCAACGCCAGCGGCG
TGGACGCCAAGGCCATCCTGTCTGCCAGACTGAGCAAGAGCAGACGGCTGGAAAATCTGATCGCCCAGCT
GCCCGGCGAGAAGAAGAATGGCCTGTTCGGAAACCTGATTGCCCTGAGCCTGGGCCTGACCCCCAACTTC
AAGAGCAACTTCGACCTGGCCGAGGATGCCAAACTGCAGCTGAGCAAGGACACCTACGACGACGACCTGG
ACAACCTGCTGGCCCAGATCGGCGACCAGTACGCCGACCTGTTTCTGGCCGCCAAGAACCTGTCCGACGC
CATCCTGCTGAGCGACATCCTGAGAGTGAACACCGAGATCACCAAGGCCCCCCTGAGCGCCTCTATGATC
AAGAGATACGACGAGCACCACCAGGACCTGACCCTGCTGAAAGCTCTCGTGCGGCAGCAGCTGCCTGAGA
AGTACAAAGAGATTTTCTTCGACCAGAGCAAGAACGGCTACGCCGGCTACATTGACGGCGGAGCCAGCCA
GGAAGAGTTCTACAAGTTCATCAAGCCCATCCTGGAAAAGATGGACGGCACCGAGGAACTGCTCGTGAAG
CTGAACAGAGAGGACCTGCTGCGGAAGCAGCGGACCTTCGACAACGGCAGCATCCCCCACCAGATCCACC
TGGGAGAGCTGCACGCCATTCTGCGGCGGCAGGAAGATTTTTACCCATTCCTGAAGGACAACCGGGAAAA
GATCGAGAAGATCCTGACCTTCCGCATCCCCTACTACGTGGGCCCTCTGGCCAGGGGAAACAGCAGATTC
GCCTGGATGACCAGAAAGAGCGAGGAAACCATCACCCCCTGGAACTTCGAGGAAGTGGTGGACAAGGGCG
CTTCCGCCCAGAGCTTCATCGAGCGGATGACCAACTTCGATAAGAACCTGCCCAACGAGAAGGTGCTGCC
CAAGCACAGCCTGCTGTACGAGTACTTCACCGTGTATAACGAGCTGACCAAAGTGAAATACGTGACCGAG
GGAATGAGAAAGCCCGCCTTCCTGAGCGGCGAGCAGAAAAAGGCCATCGTGGACCTGCTGTTCAAGACCA
ACCGGAAAGTGACCGTGAAGCAGCTGAAAGAGGACTACTTCAAGAAAATCGAGTGCTTCGACTCCGTGGA
AATCTCCGGCGTGGAAGATCGGTTCAACGCCTCCCTGGGCACATACCACGATCTGCTGAAAATTATCAAG
GACAAGGACTTCCTGGACAATGAGGAAAACGAGGACATTCTGGAAGATATCGTGCTGACCCTGACACTGT
TTGAGGACAGAGAGATGATCGAGGAACGGCTGAAAACCTATGCCCACCTGTTCGACGACAAAGTGATGAA
GCAGCTGAAGCGGCGGAGATACACCGGCTGGGGCAGGCTGAGCCGGAAGCTGATCAACGGCATCCGGGAC
AAGCAGTCCGGCAAGACAATCCTGGATTTCCTGAAGTCCGACGGCTTCGCCAACAGAAACTTCATGCAGC
TGATCCACGACGACAGCCTGACCTTTAAAGAGGACATCCAGAAAGCCCAGGTGTGCCTGTCCTACGAGAC
AGAGATCCTGACAGTGGAGTATGGCCTGCTGCCAATCGGCAAGATCGTGGAGAAGAGGATCGAGTGTACC
GTGTACTCTGTGGATAACAATGGCAACATCTATACACAGCCCGTGGCACAGTGGCACGATAGGGGAGAGC
AGGAGGTGTTCGAGTATTGCCTGGAGGACGGCAGCCTGATCAGGGCAACCAAGGACCACAAGTTCATGAC
AGTGGATGGCCAGATGCTGCCCATCGACGAGATTTTCGAGCGGGAGCTGGACCTGATGAGAGTGGATAAC
CTGCCTAATtgagaattcctagagctcgctgatcagcctcgactgtgccttctagttgccagccatctgt
tgtttgcccctcccccgtgccttccttgaccctggaaggtgccactcccactgtcctttcctaataaaat
gaggaaattgcatcgcattgtctgagtaggtgtcattctattctggggggtggggtggggcaggacagca
agggggaggattgggaagagaatagcaggcatgctggggagcggccgcaggaacccctagtgatggagtt
ggccactccctctctgcgcgctcgctcgctcactgaggccgggcgaccaaaggtcgcccgacgcccgggc
tttgcccgggcggcctcagtgagcgagcgagcgcgcagctgcctgcaggggcgcctgatgcggtattttc
tccttacgcatctgtgcggtatttcacaccgcatacgtcaaagcaaccatagtacgcgccctgtagcggc
gcattaagcgcggcgggtgtggtggttacgcgcagcgtgaccgctacacttgccagcgccctagcgcccg
ctcctttcgctttcttcccttcctttctcgccacgttcgccggctttccccgtcaagctctaaatcgggg

gctccctttagggttccgatttagtgctttacggcacctcgaccccaaaaaacttgatttgggtgatggt
tcacgtagtgggccatcgccctgatagacggtttttcgccctttgacgttggagtccacgttctttaata
gtggactcttgttccaaactggaacaacactcaaccctatctcgggctattcttttgatttataagggat
tttgccgatttcggcctattggttaaaaaatgagctgatttaacaaaaatttaacgcgaattttaacaaa
atattaacgtttacaattttatggtgcactctcagtacaatctgctctgatgccgcatagttaagccagc
cccgacacccgccaacacccgctgacgcgccctgacgggcttgtctgctcccggcatccgcttacagaca
agctgtgaccgtctccgggagctgcatgtgtcagaggttttcaccgtcatcaccgaaacgcgcgagacga
aagggcctcgtgatacgcctatttttataggttaatgtcatgataataatggtttcttagacgtcaggtg
gcacttttcggggaaatgtgcgcggaacccctatttgtttatttttctaaatacattcaaatatgtatcc
gctcatgagacaataaccctgataaatgcttcaataatattgaaaaaggaagagtatgagtattcaacat
ttccgtgtcgcccttattcccttttttgcggcattttgccttcctgtttttgctcacccagaaacgctgg
tgaaagtaaaagatgctgaagatcagttgggtgcacgagtgggttacatcgaactggatctcaacagcgg
taagatccttgagagttttcgccccgaagaacgttttccaatgatgagcacttttaaagttctgctatgt
ggcgcggtattatcccgtattgacgccgggcaagagcaactcggtcgccgcatacactattctcagaatg
acttggttgagtactcaccagtcacagaaaagcatcttacggatggcatgacagtaagagaattatgcag
tgctgccataaccatgagtgataacactgcggccaacttacttctgacaacgatcggaggaccgaaggag
ctaaccgcttttttgcacaacatgggggatcatgtaactcgccttgatcgttgggaaccggagctgaatg
aagccataccaaacgacgagcgtgacaccacgatgcctgtagcaatggcaacaacgttgcgcaaactatt
aactggcgaactacttactctagcttcccggcaacaattaatagactggatggaggcggataaagttgca
ggaccacttctgcgctcggcccttccggctggctggtttattgctgataaatctggagccggtgagcgtg
gaagccgcggtatcattgcagcactggggccagatggtaagccctcccgtatcgtagttatctacacgac
ggggagtcaggcaactatggatgaacgaaatagacagatcgctgagataggtgcctcactgattaagcat
tggtaactgtcagaccaagtttactcatatatactttagattgatttaaaacttcatttttaatttaaaa
ggatctaggtgaagatcctttttgataatctcatgaccaaaatcccttaacgtgagttttcgttccactg
agcgtcagaccccgtagaaaagatcaaaggatcttcttgagatcctttttttctgcgcgtaatctgctgc
ttgcaaacaaaaaaaccaccgctaccagcggtggtttgtttgccggatcaagagctaccaactctttttc
cgaaggtaactggcttcagcagagcgcagataccaaatactgtccttctagtgtagccgtagttaggcca
ccacttcaagaactctgtagcaccgcctacatacctcgctctgctaatcctgttaccagtggctgctgcc
agtggcgataagtcgtgtcttaccgggttggactcaagacgatagttaccggataaggcgcagcggtcgg
gctgaacggggggttcgtgcacacagcccagcttggagcgaacgacctacaccgaactgagatacctaca
gcgtgagctatgagaaagcgccacgcttcccgaagggagaaaggcggacaggtatccggtaagcggcagg
gtcggaacaggagagcgcacgagggagcttccagggggaaacgcctggtatctttatagtcctgtcgggt
ttcgccacctctgacttgagcgtcgatttttgtgatgctcgtcaggggggcggagcctatggaaaaacgc
cagcaacgcggcctttttacggttcctggccttttgctggccttttgctcacatgt

Cter-Npu-intein-SpCas9(C)- linker- M-MLV_reverse_transcriptase-BPSV40_NLS

cctgcaggcagctgcgcgctcgctcgctcactgaggccgcccgggcaaagcccgggcgtcgggcgacctt
tggtcgcccggcctcagtgagcgagcgagcgcgcagagagggagtggccaactccatcactaggggttcc
ttctagaAtggaggcggtactatgtagatgagaattcaggagcaaactgggaaaagcaactgcttccaaa
tatttgtgatttttacagtgtagttttggaaaaactcttagcctaccaattcttctaagtgttttaaaat
gtgggagccagtacacatgaagttatagagtgttttaatgaggcttaaatatttaccgtaactatgaaat
gctacgcatatcatgctgttcaggctccgtggccacgcaactcatactaccggtGCCACCATGATCAAGA
TTGCTACACGGAAATACCTGGGAAAGCAGAACGTGTACGACATCGGCGTGGAGCGGGATCACAACTTCGC
CCTGAAGAATGGCTTTATCGCCAGCAATTCCGGCCAGGGCGATAGCCTGCACGAGCACATTGCCAATCTG
GCCGGCAGCCCCGCCATTAAGAAGGGCATCCTGCAGACAGTGAAGGTGGTGGACGAGCTCGTGAAAGTGA
TGGGCCGGCACAAGCCCGAGAACATCGTGATCGAAATGGCCAGAGAGAACCAGACCACCCAGAAGGGACA

GAAGAACAGCCGCGAGAGAATGAAGCGGATCGAAGAGGGCATCAAAGAGCTGGGCAGCCAGATCCTGAAA
GAACACCCCGTGGAAAACACCCAGCTGCAGAACGAGAAGCTGTACCTGTACTACCTGCAGAATGGGCGGG
ATATGTACGTGGACCAGGAACTGGACATCAACCGGCTGTCCGACTACGATGTGGACGCTATCGTGCCTCA
GAGCTTTCTGAAGGACGACTCCATCGACAACAAGGTGCTGACCAGAAGCGACAAGAACCGGGCAAGAGC
GACAACGTGCCCTCCGAAGAGGTCGTGAAGAAGATGAAGAACTACTGGCGGCAGCTGCTGAACGCCAAGC
TGATTACCCAGAGAAAGTTCGACAATCTGACCAAGGCCGAGAGAGGCGGCCTGAGCGAACTGGATAAGGC
CGGCTTCATCAAGAGACAGCTGGTGGAAACCCGGCAGATCACAAAGCACGTGGCACAGATCCTGGACTCC
CGGATGAACACTAAGTACGACGAGAATGACAAGCTGATCCGGGAAGTGAAAGTGATCACCCTGAAGTCCA
AGCTGGTGTCCGATTTCCGGAAGGATTTCCAGTTTTACAAAGTGCGCGAGATCAACAACTACCACCACGC
CCACGACGCCTACCTGAACGCCGTCGTGGGAACCGCCCTGATCAAAAAGTACCCTAAGCTGGAAAGCGAG
TTCGTGTACGGCGACTACAAGGTGTACGACGTGCGGAAGATGATCGCCAAGAGCGAGCAGGAAATCGGCA
AGGCTACCGCCAAGTACTTCTTCTACAGCAACATCATGAACTTTTTCAAGACCGAGATTACCCTGGCCAA
CGGCGAGATCCGGAAGCGGCCTCTGATCGAGACAAACGGCGAAACCGGGGAGATCGTGTGGGATAAGGGC
CGGGATTTTGCCACCGTGCGGAAAGTGCTGAGCATGCCCCAAGTGAATATCGTGAAAAAGACCGAGGTGC
AGACAGGCGGCTTCAGCAAAGAGTCTATCCTGCCCAAGAGGAACAGCGATAAGCTGATCGCCAGAAAGAA
GGACTGGGACCCTAAGAAGTACGGCGGCTTCGACAGCCCCACCGTGGCCTATTCTGTGCTGGTGGTGGCC
AAAGTGGAAAAGGGCAAGTCCAAGAAACTGAAGAGTGTGAAAGAGCTGCTGGGGATCACCATCATGGAAA
GAAGCAGCTTCGAGAAGAATCCCATCGACTTTCTGGAAGCCAAGGGCTACAAAGAAGTGAAAAAGGACCT
GATCATCAAGCTGCCTAAGTACTCCCTGTTCGAGCTGGAAAACGGCCGGAAGAGAATGCTGGCCTCTGCC
GGCGAACTGCAGAAGGGAAACGAACTGGCCCTGCCCTCCAAATATGTGAACTTCCTGTACCTGGCCAGCC
ACTATGAGAAGCTGAAGGGCTCCCCCGAGGATAATGAGCAGAAACAGCTGTTTGTGGAACAGCACAAGCA
CTACCTGGACGAGATCATCGAGCAGATCAGCGAGTTCTCCAAGAGAGTGATCCTGGCCGACGCTAATCTG
GACAAAGTGCTGTCCGCCTACAACAAGCACCGGGATAAGCCCATCAGAGAGCAGGCCGAGAATATCATCC
ACCTGTTTACCCTGACCAATCTGGGAGCCCCTGCCGCCTTCAAGTACTTTGACACCACCATCGACCGGAA
GAGGTACACCAGCACCAAAGAGGTGCTGGACGCCACCCTGATCCACCAGAGCATCACCGGCCTGTACGAG
ACACGGATCGACCTGTCTCAGCTGGGAGGTGACTCTGGAGGATCTAGCGGAGGATCCTCTGGCAGCGAGA
CACCAGGAACAAGCGAGTCAGCAACACCAGAGAGCAGTGGCGGCAGCAGCGGCGGCAGCAGCACCCTAAA
TATAGAAGATGAGTATCGGCTACATGAGACCTCAAAAGAGCCAGATGTTTCTCTAGGGTCCACATGGCTG
TCTGATTTTCCTCAGGCCTGGGCGGAAACCGGGGGCATGGGACTGGCAGTTCGCCAAGCTCCTCTGATCA
TACCTCTGAAAGCAACCTCTACCCCCGTGTCCATAAAACAATACCCCATGTCACAAGAAGCCAGACTGGG
GATCAAGCCCCACATACAGAGACTGTTGGACCAGGGAATACTGGTACCCTGCCAGTCCCCCTGGAACACG
CCCCTGCTACCCGTTAAGAAACCAGGGACTAATGATTATAGGCCTGTCCAGGATCTGAGAGAAGTCAACA
AGCGGGTGGAAGACATCCACCCCACCGTGCCCAACCCTTACAACCTCTTGAGCGGGCTCCCACCGTCCCA
CCAGTGGTACACTGTGCTTGATTTAAAGGATGCCTTTTTCTGCCTGAGACTCCACCCCACCAGTCAGCCT
CTCTTCGCCTTTGAGTGGAGAGATCCAGAGATGGGAATCTCAGGACAATTGACCTGGACCAGACTCCCAC
AGGGTTTCAAAAACAGTCCCACCCTGTTTAATGAGGCACTGCACAGAGACCTAGCAGACTTCCGGATCCA
GCACCCAGACTTGATCCTGCTACAGTACGTGGATGACTTACTGCTGGCCGCCACTTCTGAGCTAGACTGC
CAACAAGGTACTCGGGCCCTGTTACAAACCCTAGGGAACCTCGGGTATCGGGCCTCGGCCAAGAAAGCCC
AAATTTGCCAGAAACAGGTCAAGTATCTGGGGTATCTTCTAAAAGAGGGTCAGAGATGGCTGACTGAGGC
CAGAAAAGAGACTGTGATGGGGCAGCCTACTCCGAAGACCCCTCGACAACTAAGGGAGTTCCTAGGGAAG
GCAGGCTTCTGTCGCCTCTTCATCCCTGGGTTTGCAGAAATGGCAGCCCCCCTGTACCCTCTCACCAAAC
CGGGGACTCTGTTTAATTGGGGCCCAGACCAACAAAAGGCCTATCAAGAAATCAAGCAAGCTCTTCTAAC
TGCCCCAGCCCTGGGGTTGCCAGATTTGACTAAGCCCTTTGAACTCTTTGTCGACGAGAAGCAGGGCTAC
GCCAAAGGTGTCCTAACGCAAAAACTGGGACCTTGGCGTCGGCCGGTGGCCTACCTGTCCAAAAAGCTAG
ACCCAGTAGCAGCTGGGTGGCCCCCTTGCCTACGGATGGTAGCAGCCATTGCCGTACTGACAAAGGATGC
AGGCAAGCTAACCATGGACAGCCACTAGTCATTCTGGCCCCCCATGCAGTAGAGGCACTAGTCAAACAA
CCCCCCGACCGCTGGCTTTCCAACGCCCGGATGACTCACTATCAGGCCTTGCTTTTGGACACGGACCGGG
TCCAGTTCGGACCGGTGGTAGCCCTGAACCCGGCTACGCTGCTCCCACTGCCTGAGGAAGGGCTGCAACA

CAACTGCCTTGATATCCTGGCCGAAGCCCACGGAACCCGACCCGACCTAACGGACCAGCCGCTCCCAGAC
GCCGACCACACCTGGTACACGGATGGAAGCAGTCTCTTACAAGAGGGACAGCGTAAGGCGGGAGCTGCGG
TGACCACCGAGACCGAGGTAATCTGGGCTAAAGCCCTGCCAGCCGGGACATCCGCTCAGCGGGCTGAACT
GATAGCACTCACCCAGGCCCTAAAGATGGCAGAAGGTAAGAAGCTAAATGTTTATACTGATAGCCGTTAT
GCTTTTGCTACTGCCCATATCCATGGAGAAATATACAGAAGGCGTGGGTGGCTCACATCAGAAGGCAAAG
AGATCAAAAATAAAGACGAGATCTTGGCCCTACTAAAAGCCCTCTTTCTGCCCAAAAGACTTAGCATAAT
CCATTGTCCAGGACATCAAAAGGGACACAGCGCCGAGGCTAGAGGCAACCGGATGGCTGACCAAGCGGCC
CGAAAGGCAGCCATCACAGAGACTCCAGACACCTCTACCCTCCTCATAGAAAATTCATCACCCTCTGGCG
GCTCAAAAAGAACCGCCGACGGCAGCGAATTCGAGCCCAAGAAGAAGAGGAAAGTCTAAgaaaataaagg
aaatttattttcattgcaatagtgtgttggaattttttgtgtctctcagcggccgcaggaacccctagtg
atggagttggccactccctctctgcgcgctcgctcgctcactgaggccgggcgaccaaaggtcgcccgac
gcccgggctttgcccgggcggcctcagtgagcgagcgagcgcgcagctgcctgcaggggcgcctgatgcg
gtattttctccttacgcatctgtgcggtatttcacaccgcatacgtcaaagcaaccatagtacgcgccct
gtagcggcgcattaagcgcggcgggtgtggtggttacgcgcagcgtgaccgctacacttgccagcgccct
agcgcccgctcctttcgctttcttcccttcctttctcgccacgttcgccggctttccccgtcaagctcta
aatcgggggctccctttagggttccgatttagtgctttacggcacctcgaccccaaaaaacttgatttgg
gtgatggttcacgtagtgggccatcgccctgatagacggtttttcgccctttgacgttggagtccacgtt
ctttaatagtggactcttgttccaaactggaacaacactcaaccctatctcgggctattcttttgattta
taagggattttgccgatttcggcctattggttaaaaaatgagctgatttaacaaaaatttaacgcgaatt
ttaacaaaatattaacgtttacaattttatggtgcactctcagtacaatctgctctgatgccgcatagtt
aagccagccccgacacccgccaacacccgctgacgcgccctgacgggcttgtctgctcccggcatccgct
tacagacaagctgtgaccgtctccgggagctgcatgtgtcagaggttttcaccgtcatcaccgaaacgcg
cgagacgaaagggcctcgtgatacgcctatttttataggttaatgtcatgataataatggtttcttagac
gtcaggtggcacttttcggggaaatgtgcgcggaacccctatttgtttatttttctaaatacattcaaat
atgtatccgctcatgagacaataaccctgataaatgcttcaataatattgaaaaaggaagagtatgagta
ttcaacatttccgtgtcgcccttattcccttttttgcggcattttgccttcctgtttttgctcacccaga
aacgctggtgaaagtaaaagatgctgaagatcagttgggtgcacgagtgggttacatcgaactggatctc
aacagcggtaagatccttgagagttttcgccccgaagaacgttttccaatgatgagcacttttaaagttc
tgctatgtggcgcggtattatcccgtattgacgccgggcaagagcaactcggtcgccgcatacactattc
tcagaatgacttggttgagtactcaccagtcacagaaaagcatcttacggatggcatgacagtaagagaa
ttatgcagtgctgccataaccatgagtgataacactgcggccaacttacttctgacaacgatcggaggac
cgaaggagctaaccgcttttttgcacaacatgggggatcatgtaactcgccttgatcgttgggaaccgga
gctgaatgaagccataccaaacgacgagcgtgacaccacgatgcctgtagcaatggcaacaacgttgcgc
aaactattaactggcgaactacttactctagcttcccggcaacaattaatagactggatggaggcggata
aagttgcaggaccacttctgcgctcggcccttccggctggctggtttattgctgataaatctggagccgg
tgagcgtggaagccgcggtatcattgcagcactggggccagatggtaagccctcccgtatcgtagttatc
tacacgacggggagtcaggcaactatggatgaacgaaatagacagatcgctgagataggtgcctcactga
ttaagcattggtaactgtcagaccaagtttactcatatatactttagattgatttaaaacttcatttta
atttaaaaggatctaggtgaagatcctttttgataatctcatgaccaaaatcccttaacgtgagttttcg
ttccactgagcgtcagaccccgtagaaaagatcaaaggatcttcttgagatcctttttttctgcgcgtaa
tctgctgcttgcaaacaaaaaaaccaccgctaccagcggtggtttgtttgccggatcaagagctaccaac
tctttttccgaaggtaactggcttcagcagagcgcagataccaaatactgtccttctagtgtagccgtag
ttaggccaccacttcaagaactctgtagcaccgcctacatacctcgctctgctaatcctgttaccagtgg
ctgctgccagtggcgataagtcgtgtcttaccgggttggactcaagacgatagttaccggataaggcgca
gcggtcgggctgaacggggggttcgtgcacacagcccagcttggagcgaacgacctacaccgaactgaga
tacctacagcgtgagctatgagaaagcgccacgcttcccgaagggagaaaggcggacaggtatccggtaa
gcggcagggtcggaacaggagagcgcacgagggagcttccagggggaaacgcctggtatctttatagtcc

tgtcgggtttcgccacctctgacttgagcgtcgatttttgtgatgctcgtcaggggggcggagcctatgg
aaaaacgccagcaacgcggcctttttacggttcctggccttttgctggccttttgctcacatgt

Supplementary Note 1. FACS gating examples for GFP-positive or Cherry-positive cells.

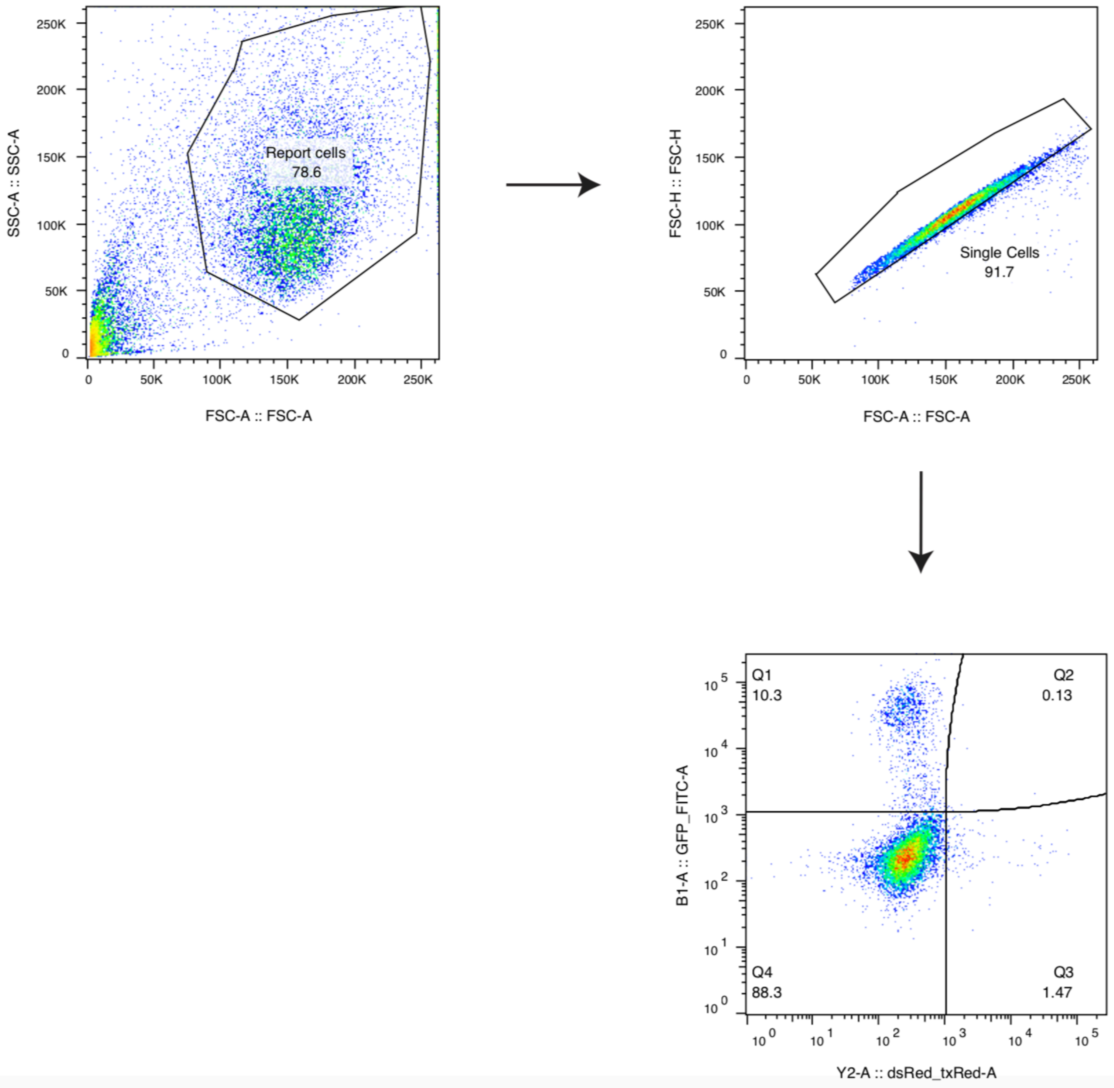

Supplement: Supplementary file 2 — Supplementary Information [file 41467_2021_22295_MOESM2_ESM.pdf]
